# Supplementary material for: Homocouplings of Sodium Arenesulfinates: Selective Access to Symmetric Diaryl Sulfides and Diaryl Disulfides
Source: Molecules. 2022 Sep 22;27(19):6232. doi: 10.3390/molecules27196232 (PMC9571168; doi:10.3390/molecules27196232)
Supplement: Supplementary file 1 [file molecules-27-06232-s001.zip › molecules-1908778-supplementary.pdf]

Supporting Information

Homocouplings of Sodium Arenesulfinates: Selective Access to Symmetric Diaryl Sulfides and Diaryl Disulfides

Xin-Zhang Yu [1,2], Wen-Long Wei [2], Yu-Lan Niu [1,*], Xing Li [2], Ming Wang [3] and Wen-Chao Gao [2,3,*]

[1] Department of Chemistry and Chemical Engineering, Taiyuan Institute of Technology, Taiyuan 030008, China

[2] Department of Biomedical Engineering, Taiyuan University of Technology, Taiyuan 030024, China

[3] School of Chemistry and Molecular Engineering, East China Normal University, 3663,

. Shanghai 200062, China

^{1}H NMR, ^{13}C NMR and ^{19}F NMR spectra

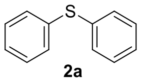

2a

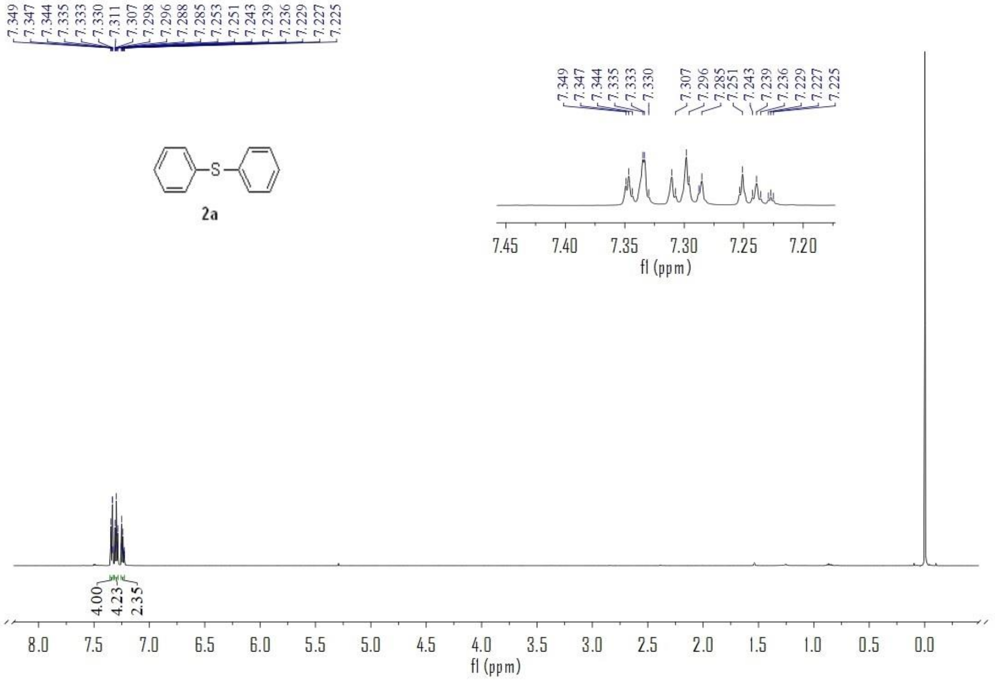

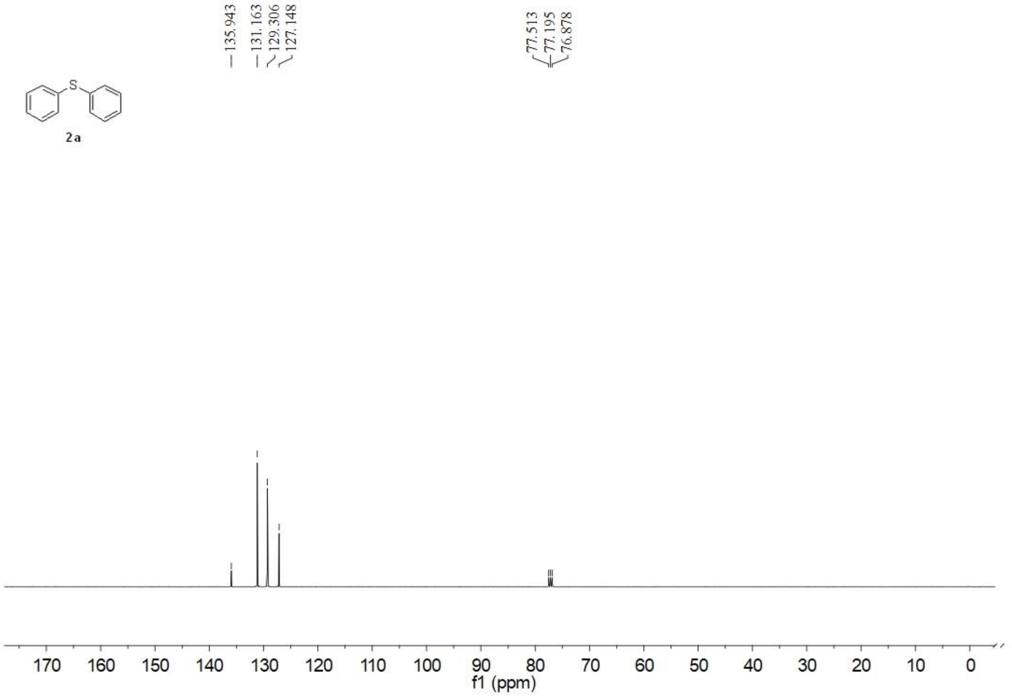

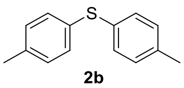

2b

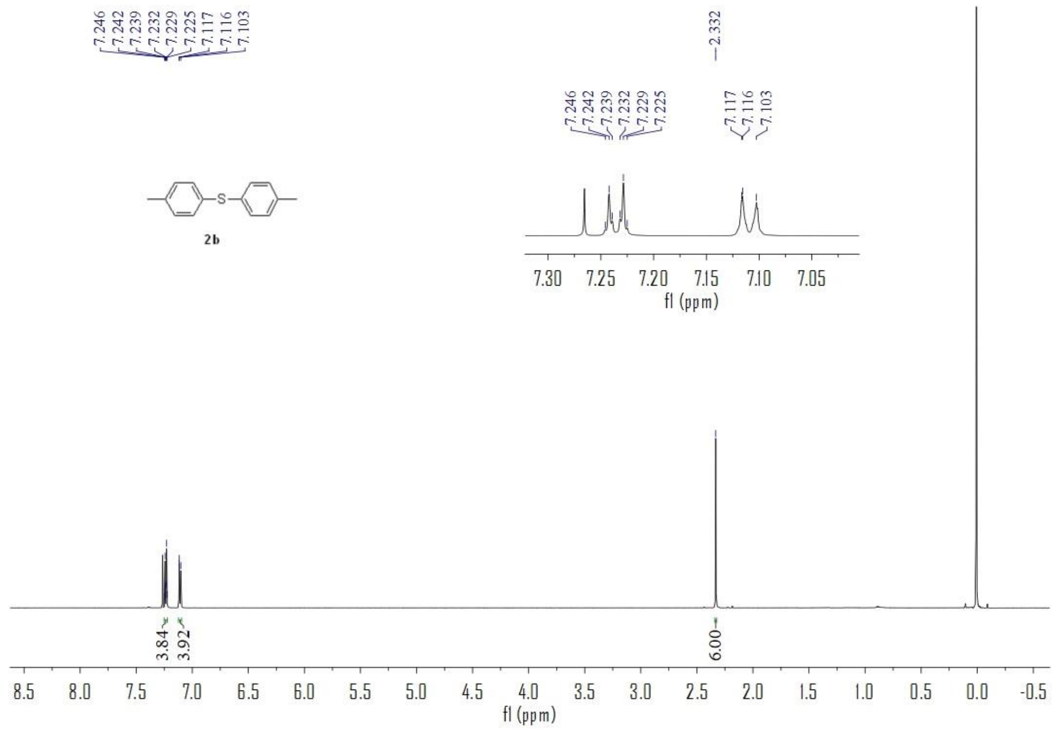
7.246
7.242
7.239
7.232
7.229
7.225
7.117
7.116
7.103
2.332
S
2b
7.30 7.25 7.20 7.15 7.10 7.05
fl (ppm)
3.84
3.92
6.00
8.5 8.0 7.5 7.0 6.5 6.0 5.5 5.0 4.5 4.0 3.5 3.0 2.5 2.0 1.5 1.0 0.5 0.0 -0.5
fl (ppm)

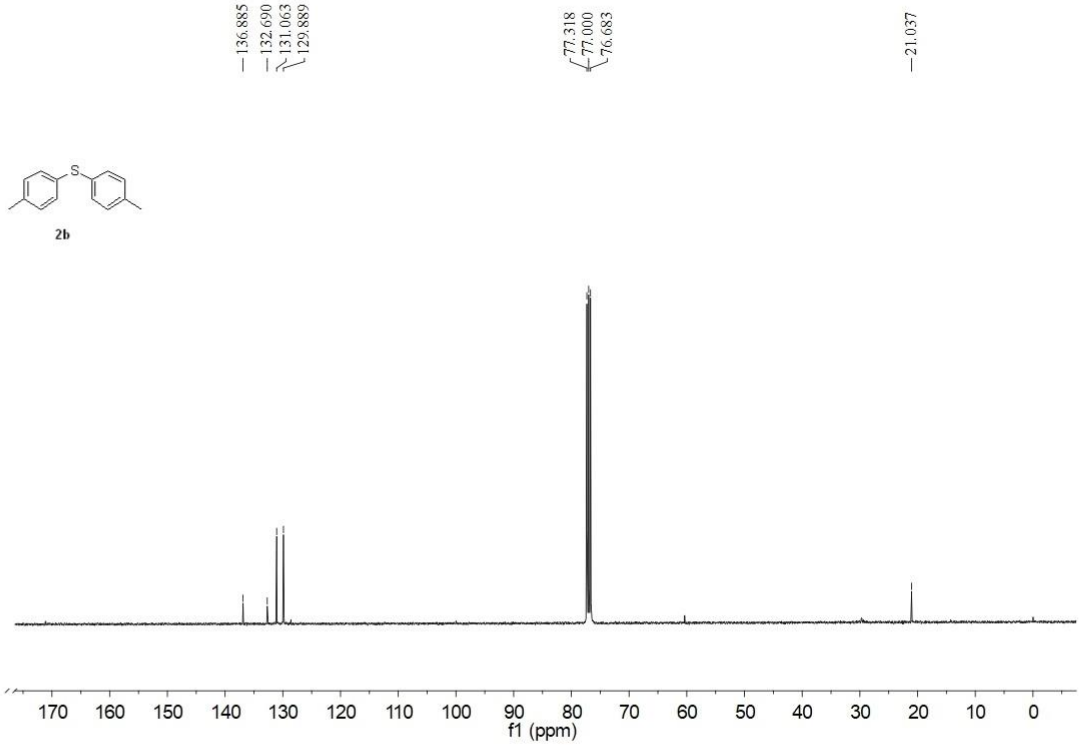
136.885
132.690
131.063
129.889
77.318
77.000
76.683
21.037
S
2b
170 160 150 140 130 120 110 100 90 80 70 60 50 40 30 20 10 0
f1 (ppm)

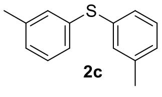

2c

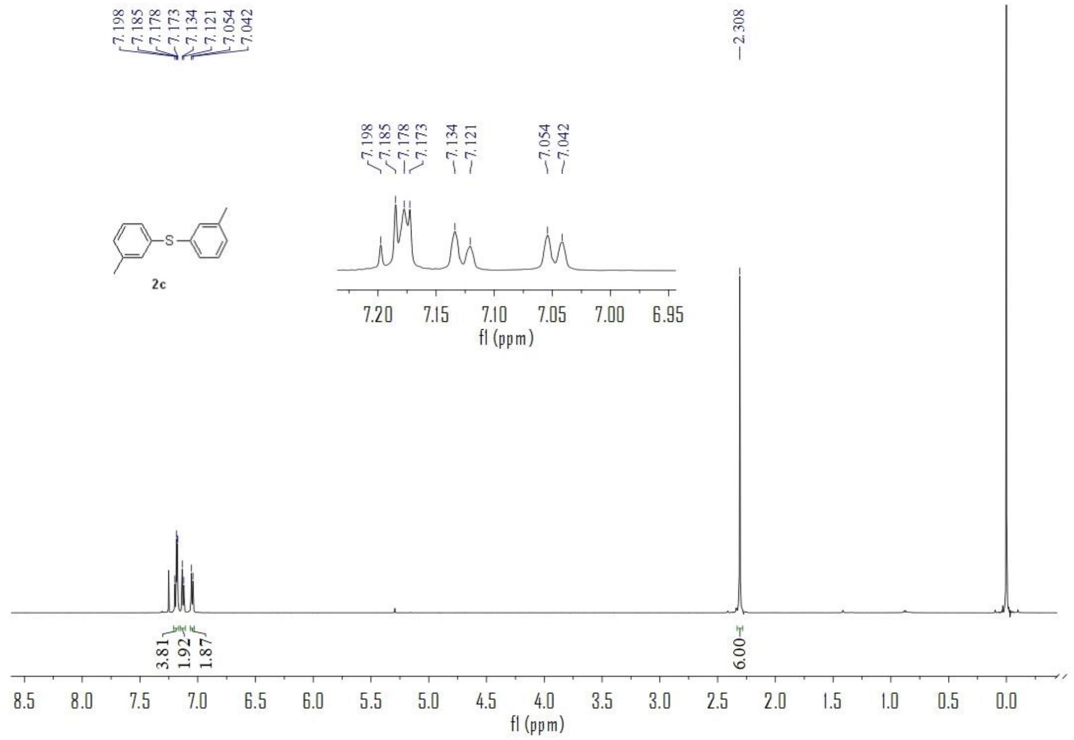
7.198
7.185
7.178
7.173
7.134
7.121
7.054
7.042
2.308
2c
3.81
1.92
1.87
6.00
fl (ppm)

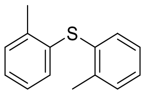

2d

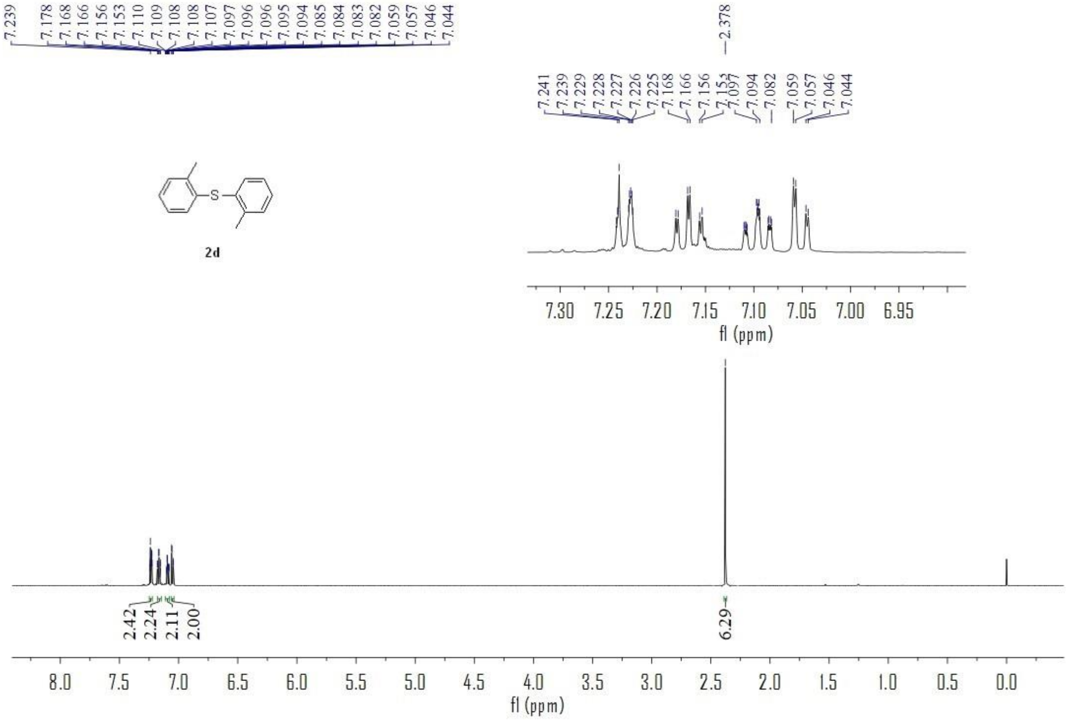
7.239
7.178
7.168
7.166
7.156
7.153
7.110
7.109
7.108
7.108
7.107
7.097
7.096
7.096
7.095
7.094
7.085
7.084
7.084
7.083
7.082
7.059
7.057
7.046
7.044
2.378
2d
2.42
2.24
2.11
2.00
6.29
fl (ppm)

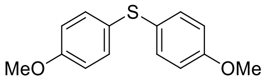
MeO
OMe

2e

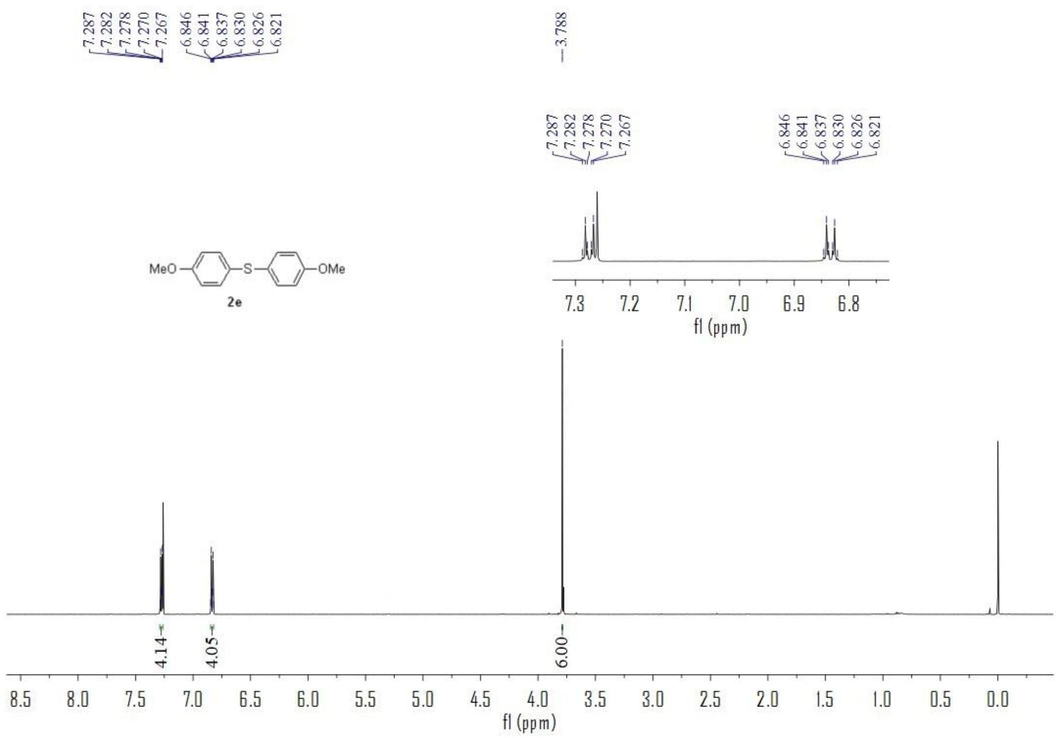
MeO
OMe
S
2e
3.788
4.14
4.05
6.00
fl (ppm)

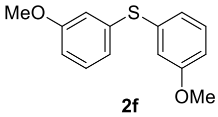

2f

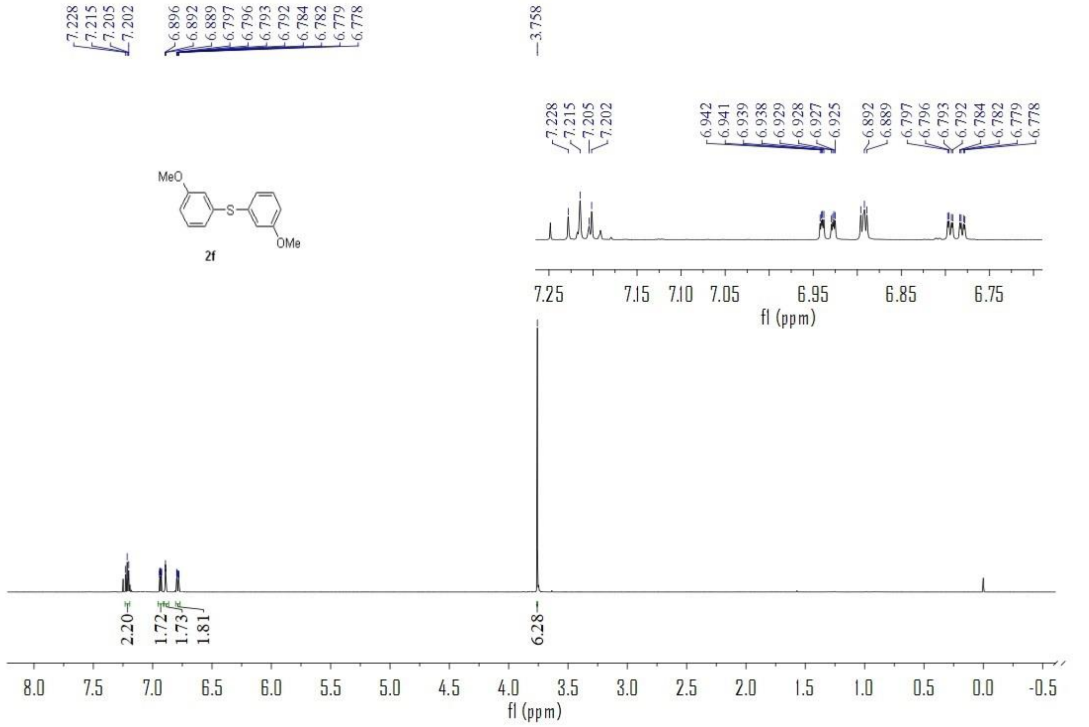
MeO
S
OMe
2f
3.758
2.20
1.72
1.73
1.81
6.28
fl (ppm)

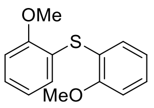
OMe
S
MeO

2g

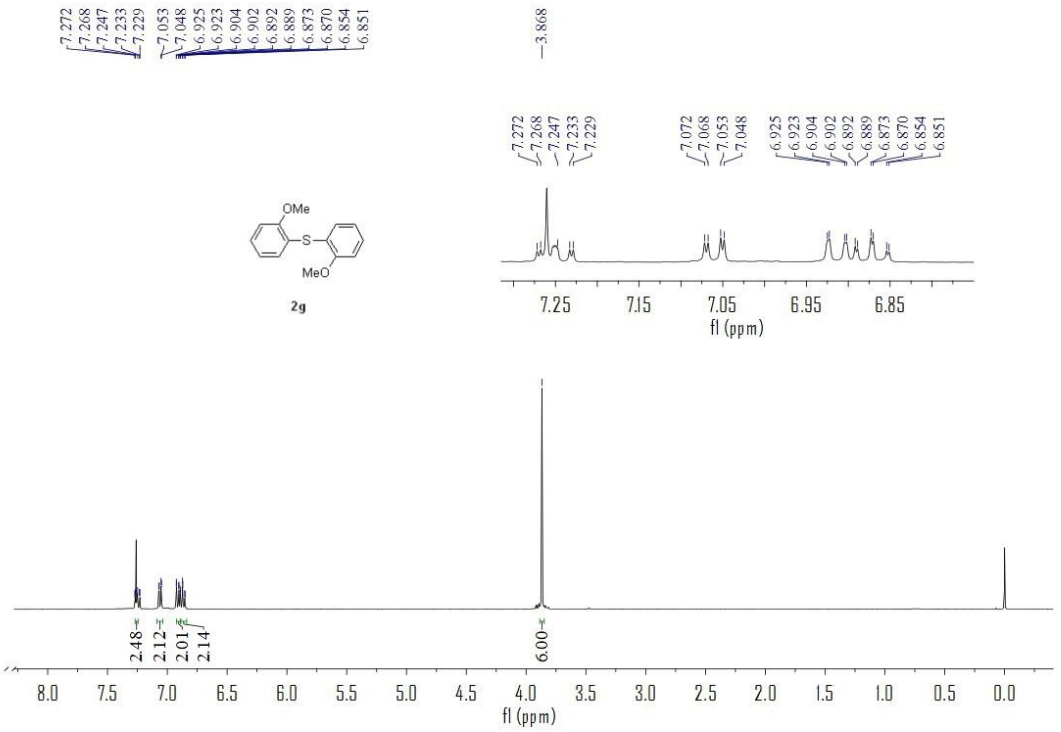
7.272 7.268 7.247 7.233 7.229 7.053 7.048 6.925 6.923 6.904 6.902 6.892 6.889 6.873 6.870 6.854 6.851
3.868
7.272 7.268 7.247 7.233 7.229
7.072 7.068 7.053 7.048
6.925 6.923 6.904 6.902 6.892 6.889 6.873 6.870 6.854 6.851
OMe
S
MeO
2g
7.25 7.15 7.05 6.95 6.85
fl (ppm)
2.48 2.12 2.01 2.14
6.00
8.0 7.5 7.0 6.5 6.0 5.5 5.0 4.5 4.0 3.5 3.0 2.5 2.0 1.5 1.0 0.5 0.0
fl (ppm)

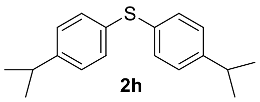
S

2h

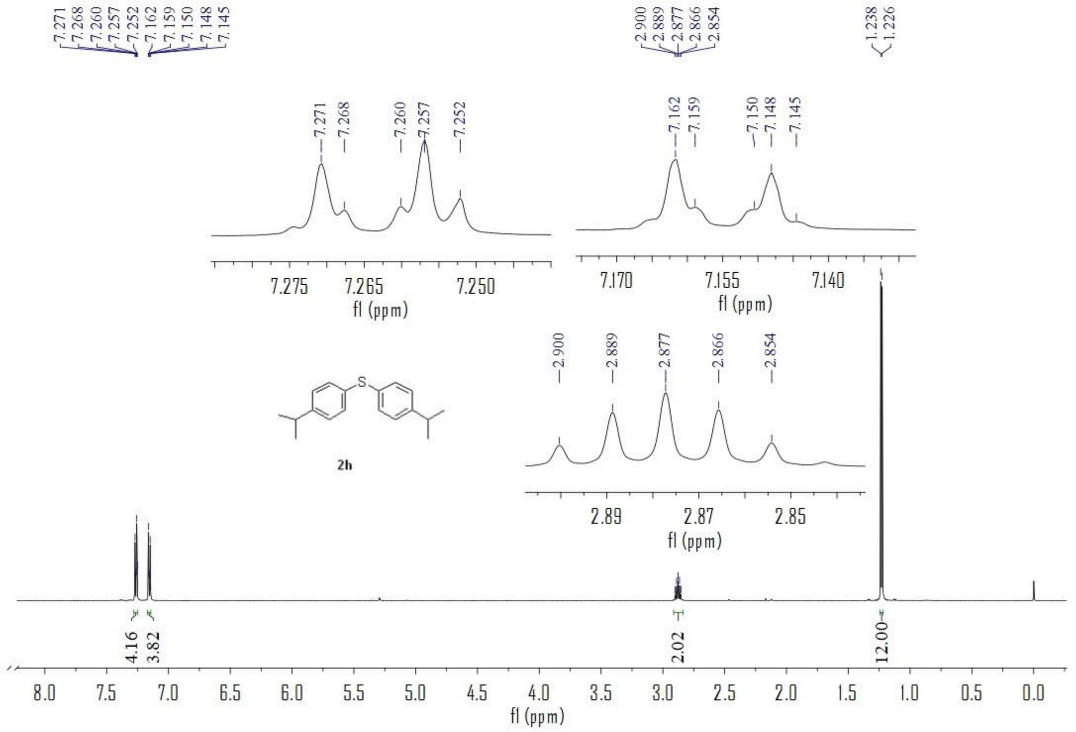
7.271 7.268 7.260 7.257 7.252 7.162 7.159 7.150 7.148 7.145
2.900 2.889 2.877 2.866 2.854
1.238 1.226
7.271 7.268 7.260 7.257 7.252
7.275 7.265 7.250
fl (ppm)
7.162 7.159 7.150 7.148 7.145
7.170 7.155 7.140
fl (ppm)
2.900 2.889 2.877 2.866 2.854
2.89 2.87 2.85
fl (ppm)
S
2h
4.16 3.82
2.02
12.00
8.0 7.5 7.0 6.5 6.0 5.5 5.0 4.5 4.0 3.5 3.0 2.5 2.0 1.5 1.0 0.5 0.0
fl (ppm)

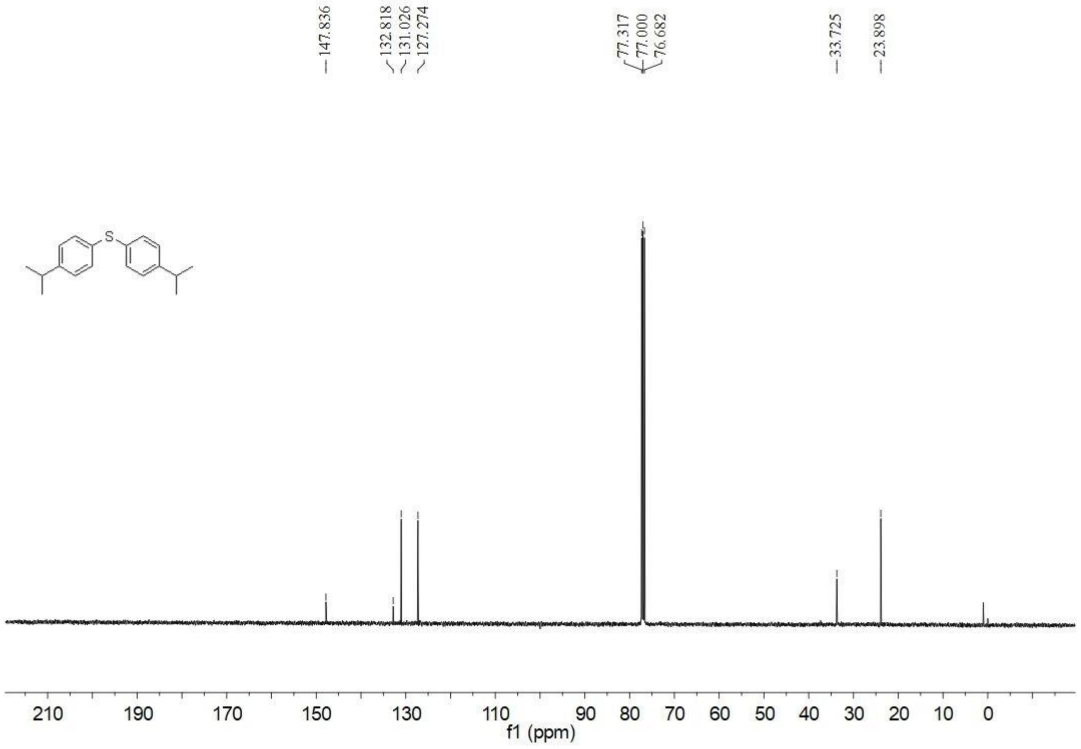
147.836
132.818
131.026
127.274
77.317
77.000
76.682
33.725
23.898
210 190 170 150 130 110 90 80 70 60 50 40 30 20 10 0
f1 (ppm)

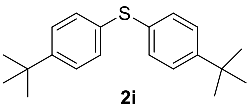

2i

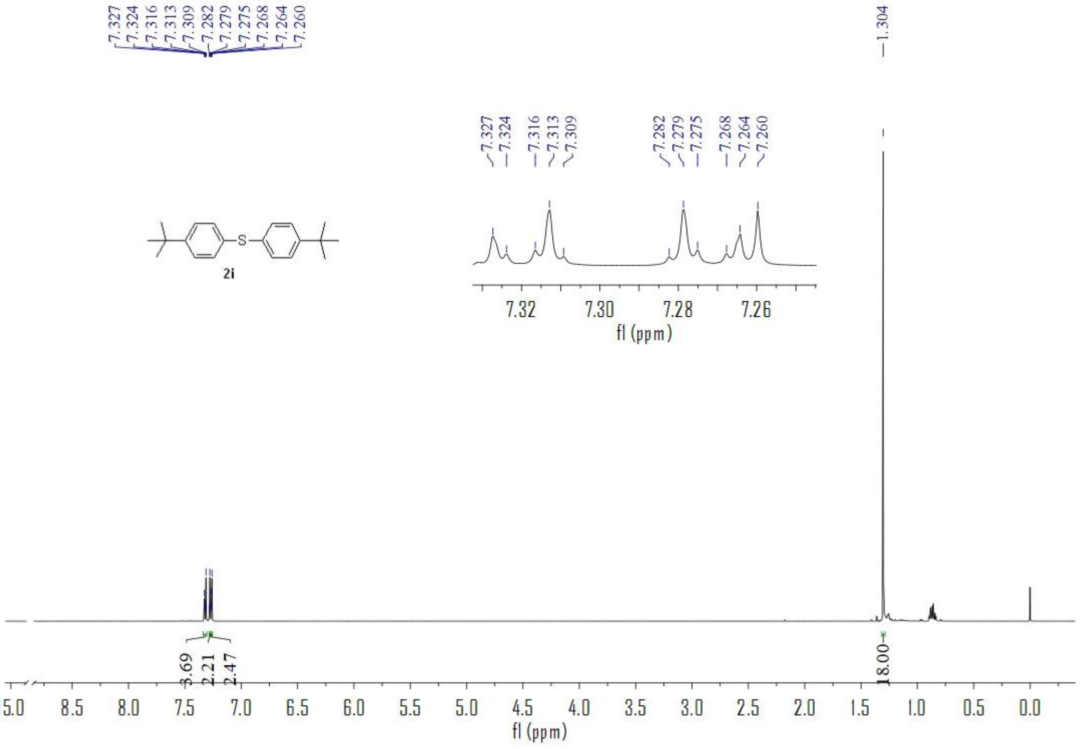
7.327
7.324
7.316
7.313
7.309
7.282
7.279
7.275
7.268
7.264
7.260
1.304
2i
7.32 7.30 7.28 7.26
fl (ppm)
3.69
2.21
2.47
18.00
15.0 8.5 8.0 7.5 7.0 6.5 6.0 5.5 5.0 4.5 4.0 3.5 3.0 2.5 2.0 1.5 1.0 0.5 0.0
fl (ppm)

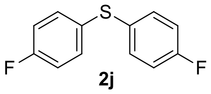
S
F
F

2j

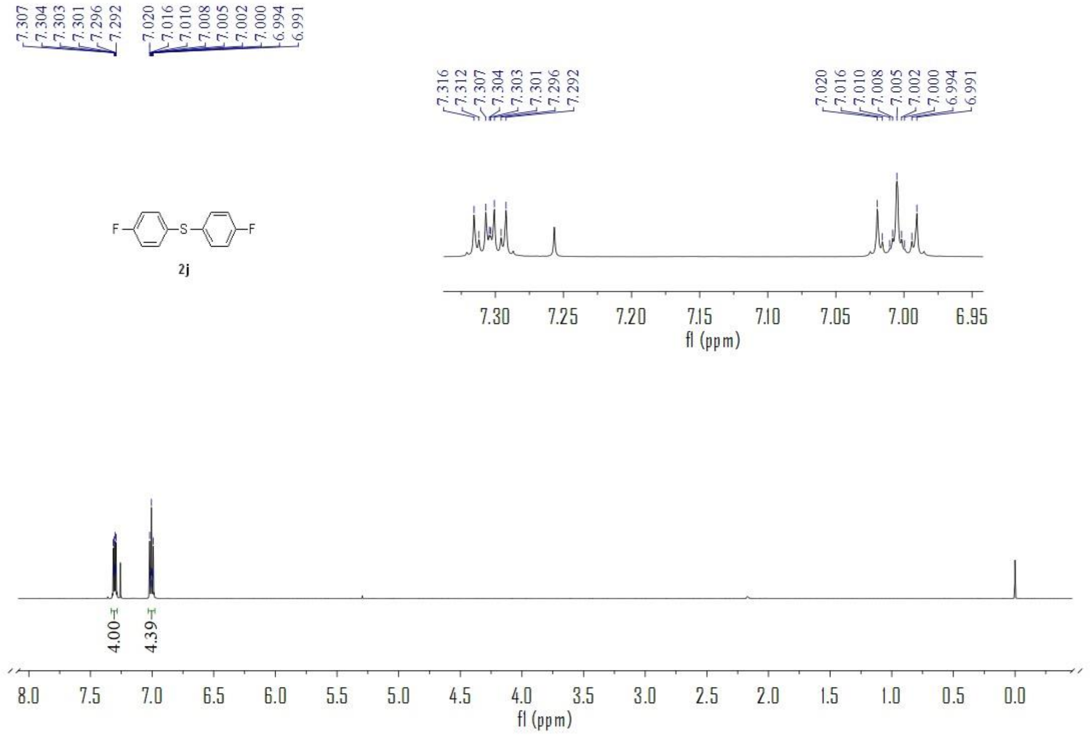
7.307
7.304
7.303
7.301
7.296
7.292
7.020
7.016
7.010
7.008
7.005
7.002
7.000
6.994
6.991
2j
7.316
7.312
7.307
7.304
7.303
7.301
7.296
7.292
7.020
7.016
7.010
7.008
7.005
7.002
7.000
6.994
6.991
7.30 7.25 7.20 7.15 7.10 7.05 7.00 6.95
fl (ppm)
4.00
4.39
8.0 7.5 7.0 6.5 6.0 5.5 5.0 4.5 4.0 3.5 3.0 2.5 2.0 1.5 1.0 0.5 0.0
fl (ppm)

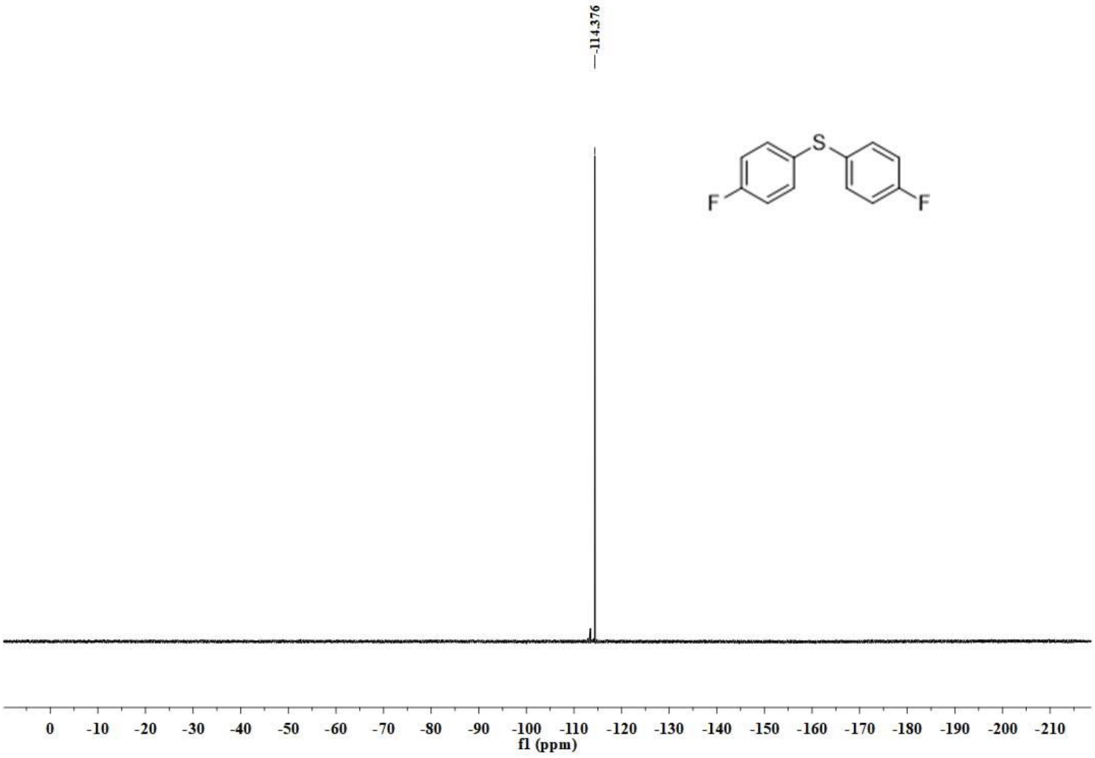
-114.376
S
F
F
0 -10 -20 -30 -40 -50 -60 -70 -80 -90 -100 -110 -120 -130 -140 -150 -160 -170 -180 -190 -200 -210
fl (ppm)

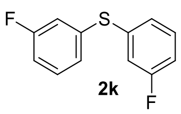
F
S
F

2k

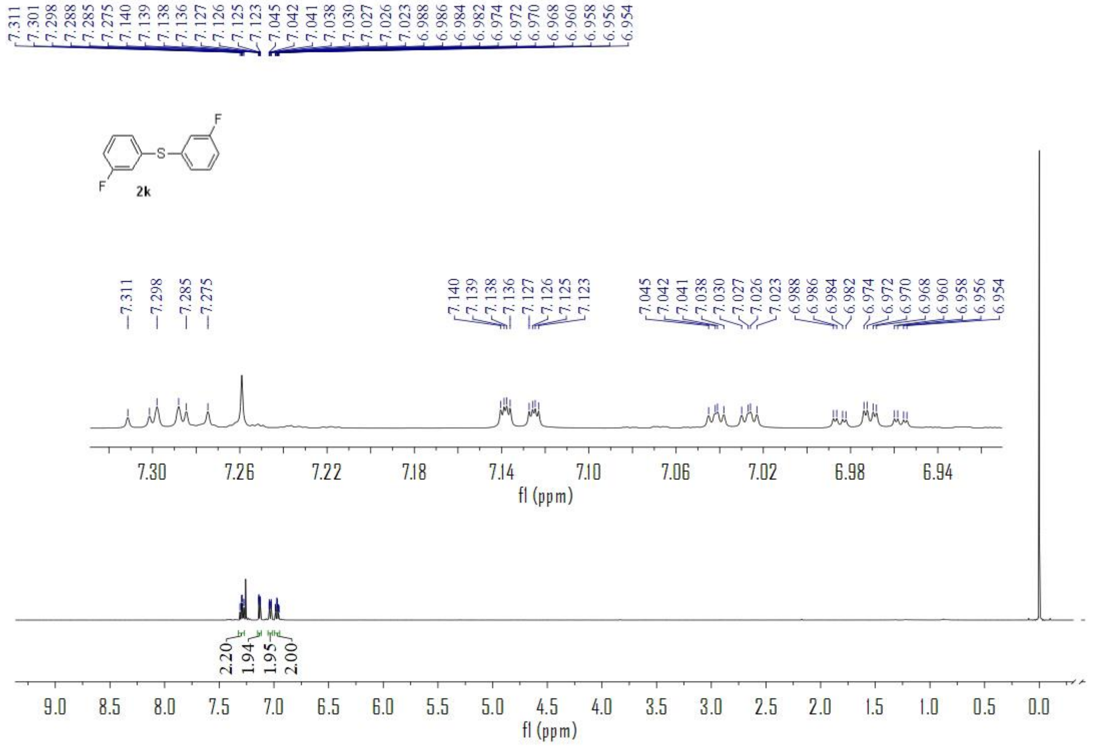
7.311
7.301
7.298
7.288
7.285
7.275
7.140
7.139
7.138
7.136
7.127
7.126
7.125
7.123
7.045
7.042
7.041
7.038
7.030
7.027
7.026
7.023
6.988
6.986
6.984
6.982
6.974
6.972
6.970
6.968
6.960
6.958
6.956
6.954
2k
2.20
1.94
1.95
2.00
f1 (ppm)

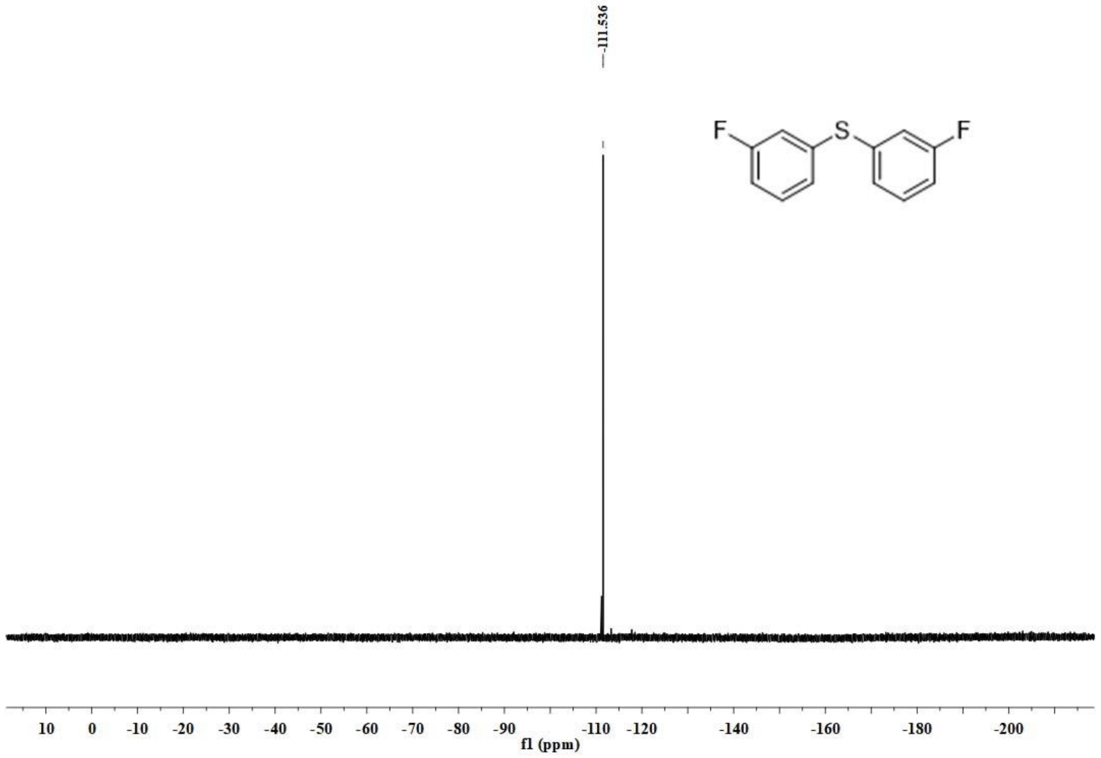
-111.536
F
S
F
f1 (ppm)

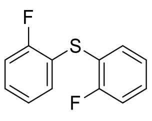

2l

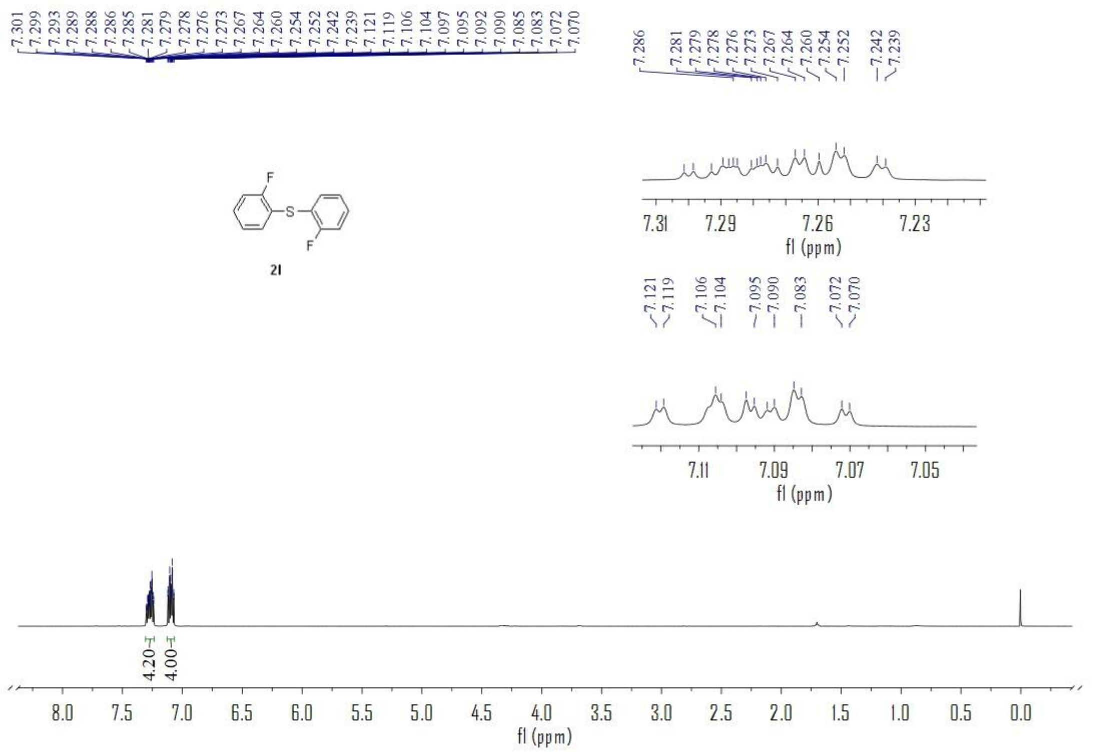
7.301 7.299 7.293 7.289 7.288 7.286 7.285 7.281 7.279 7.278 7.276 7.273 7.267 7.264 7.260 7.254 7.252 7.242 7.239 7.121 7.119 7.106 7.104 7.097 7.095 7.092 7.090 7.085 7.083 7.072 7.070
2l
7.286 7.281 7.279 7.278 7.276 7.273 7.267 7.264 7.260 7.254 7.252 7.242 7.239
7.31 7.29 7.26 7.23
fl (ppm)
7.121 7.119 7.106 7.104 7.095 7.090 7.083 7.072 7.070
7.11 7.09 7.07 7.05
fl (ppm)
4.20
4.00
8.0 7.5 7.0 6.5 6.0 5.5 5.0 4.5 4.0 3.5 3.0 2.5 2.0 1.5 1.0 0.5 0.0
fl (ppm)

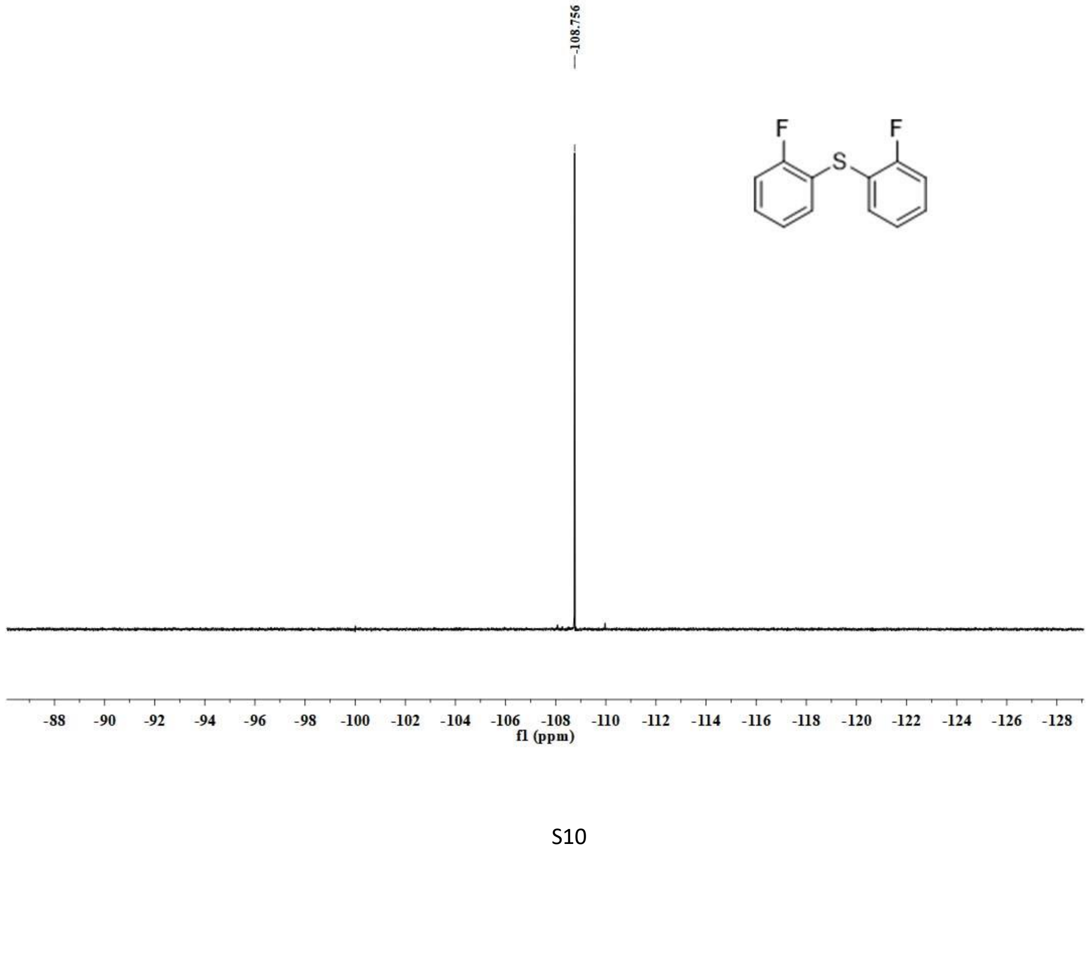
-108.756
-88 -90 -92 -94 -96 -98 -100 -102 -104 -106 -108 -110 -112 -114 -116 -118 -120 -122 -124 -126 -128
fl (ppm)

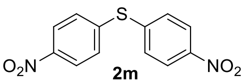

2m

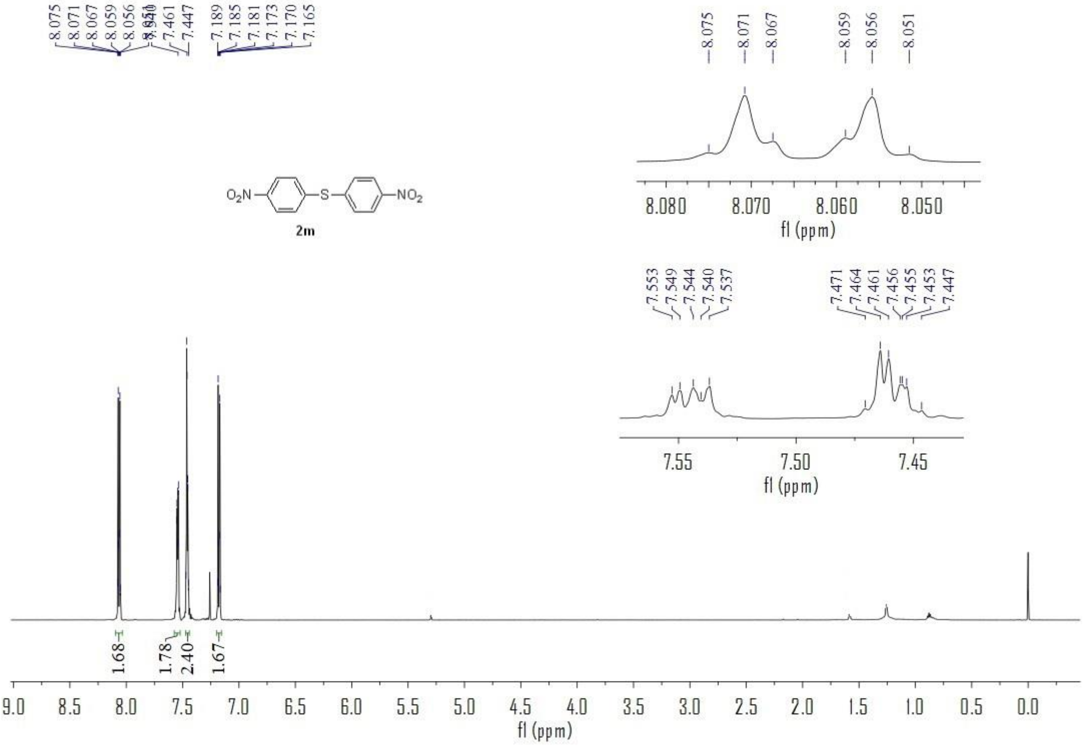

8.075
8.071
8.067
8.059
8.056
7.461
7.447
7.189
7.185
7.181
7.173
7.170
7.165
2m
8.080
8.070
8.060
8.050
fl (ppm)
7.553
7.549
7.544
7.540
7.537
7.471
7.464
7.461
7.456
7.455
7.453
7.447
7.55
7.50
7.45
fl (ppm)
1.68
1.78
2.40
1.67
9.0 8.5 8.0 7.5 7.0 6.5 6.0 5.5 5.0 4.5 4.0 3.5 3.0 2.5 2.0 1.5 1.0 0.5 0.0
fl (ppm)

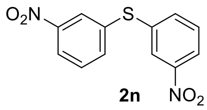

2n

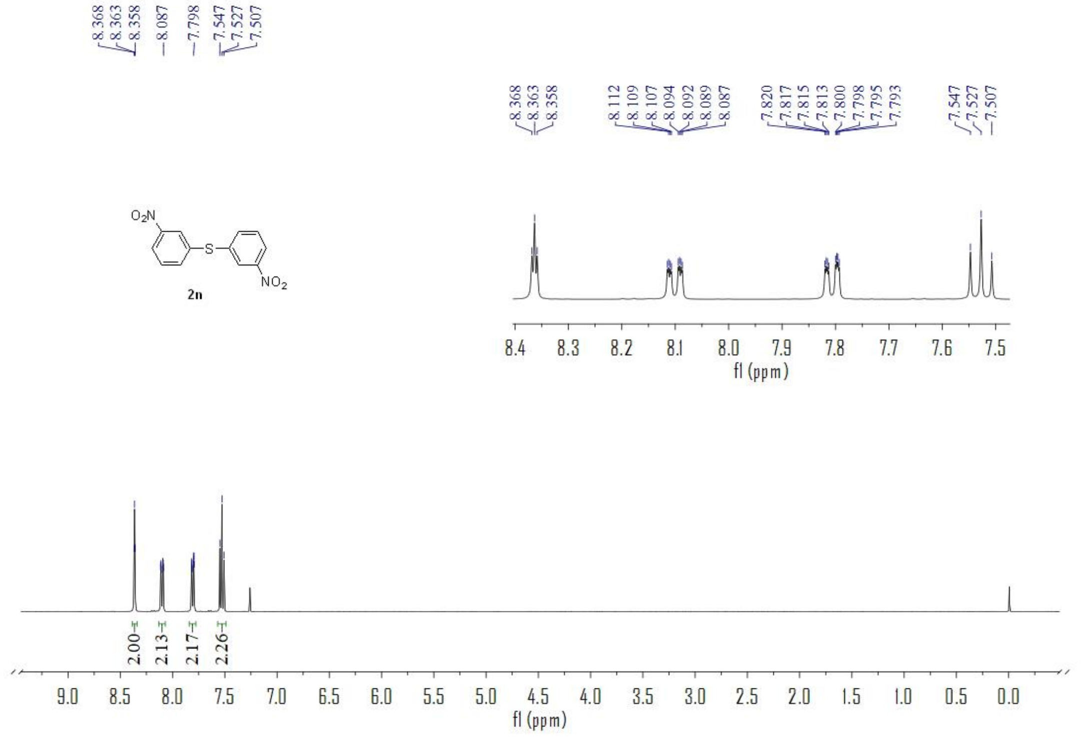

8.368
8.363
8.358
8.087
7.798
7.547
7.527
7.507
8.368
8.363
8.358
8.112
8.109
8.107
8.094
8.092
8.089
8.087
7.820
7.817
7.815
7.813
7.800
7.798
7.795
7.793
7.547
7.527
7.507
2n
8.4 8.3 8.2 8.1 8.0 7.9 7.8 7.7 7.6 7.5
fl (ppm)
2.00
2.13
2.17
2.26
9.0 8.5 8.0 7.5 7.0 6.5 6.0 5.5 5.0 4.5 4.0 3.5 3.0 2.5 2.0 1.5 1.0 0.5 0.0
fl (ppm)

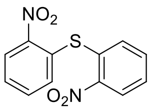

2o

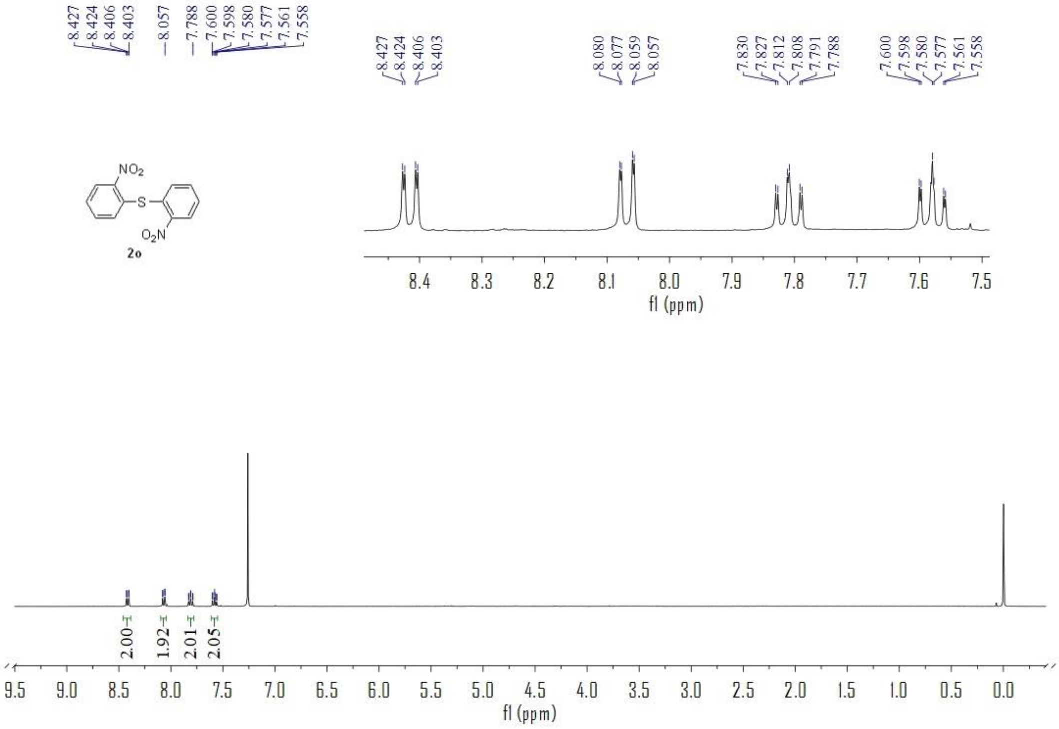

8.427
8.424
8.406
8.403
8.057
7.788
7.600
7.598
7.580
7.577
7.561
7.558
2.00
1.92
2.01
2.05
fl (ppm)

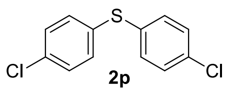

2p

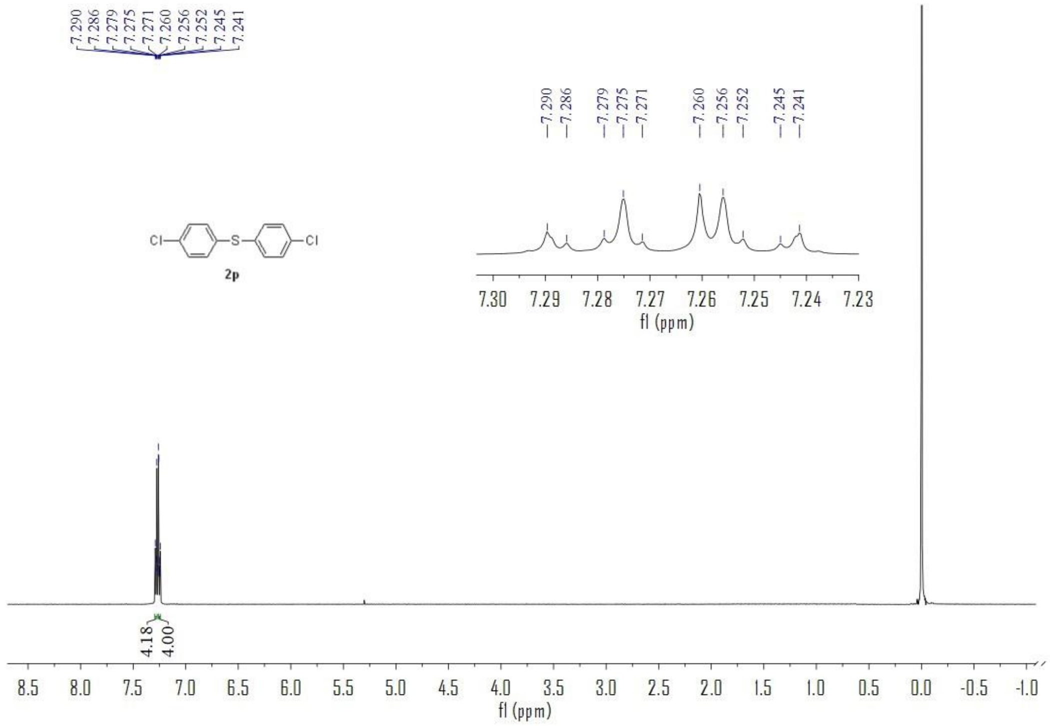

7.290
7.286
7.279
7.275
7.271
7.260
7.256
7.252
7.245
7.241
4.18
4.00
fl (ppm)

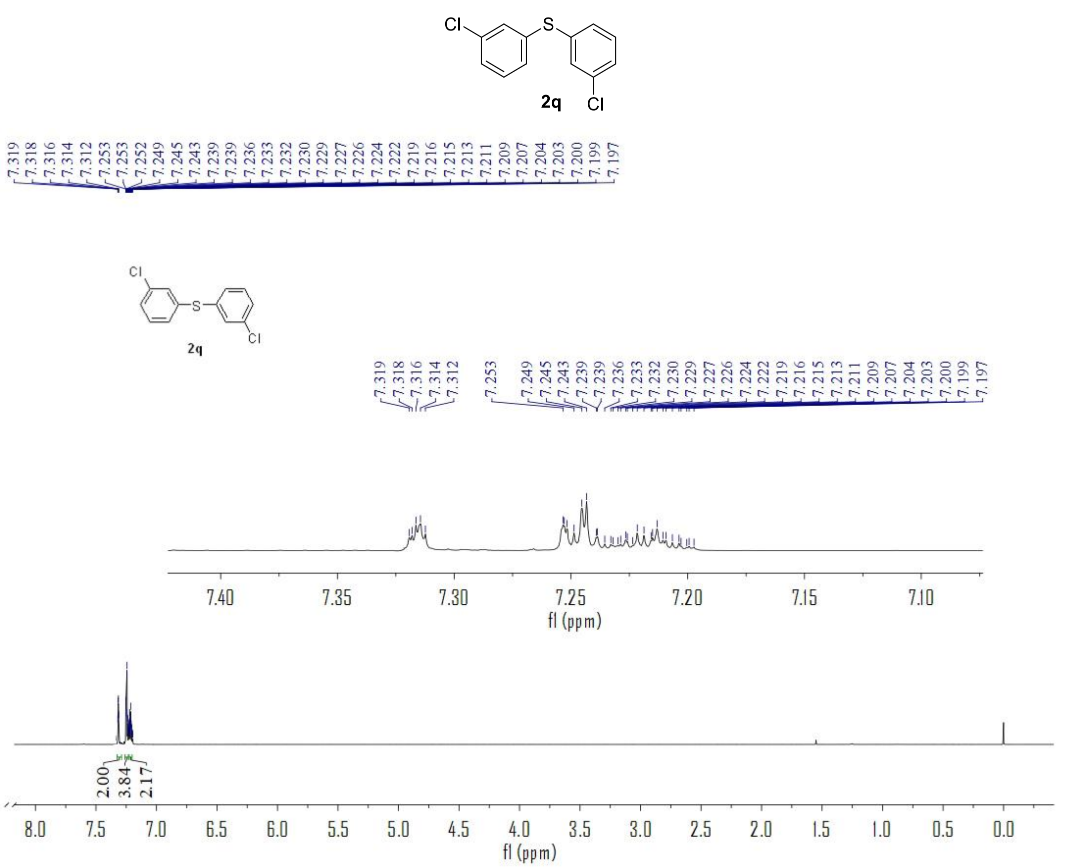
2q
7.319 7.318 7.316 7.314 7.312 7.253 7.253 7.252 7.249 7.245 7.243 7.239 7.239 7.236 7.233 7.232 7.230 7.229 7.227 7.226 7.224 7.222 7.219 7.216 7.215 7.213 7.211 7.209 7.207 7.204 7.203 7.200 7.199 7.197
2.00 3.84 2.17
fl (ppm)

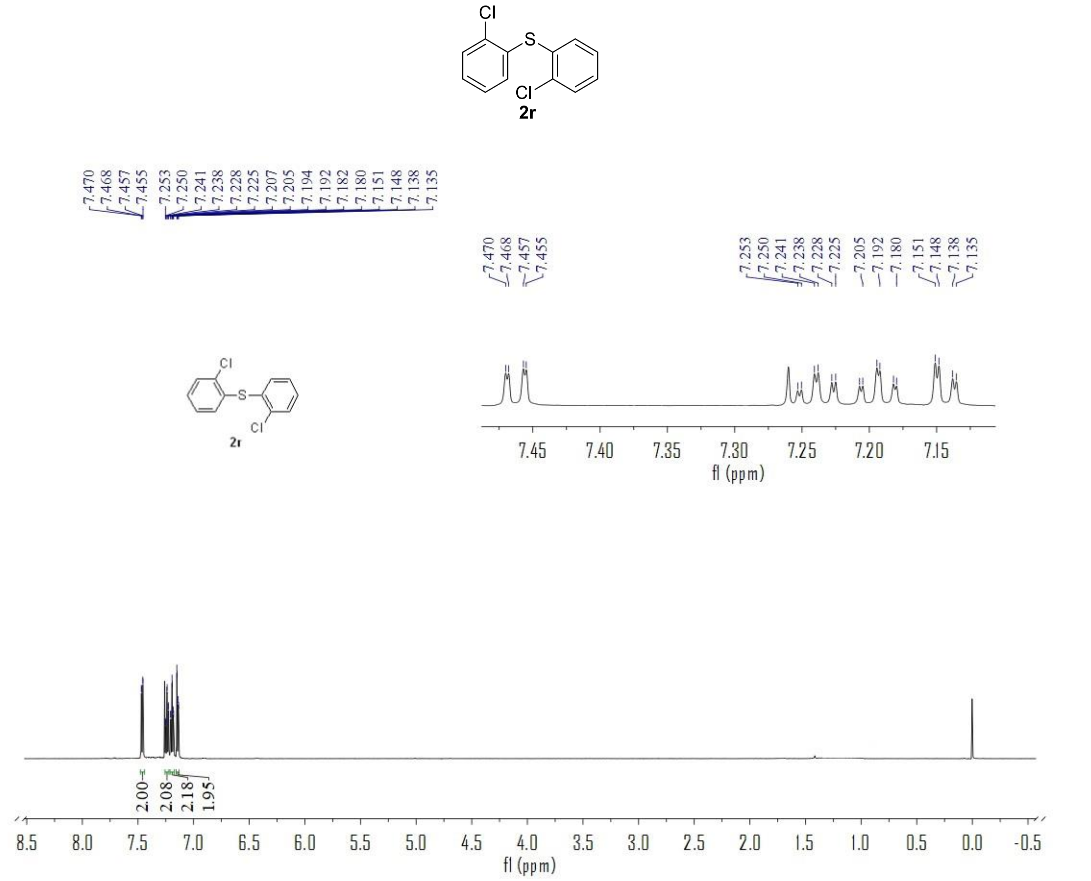
2r
7.470 7.468 7.457 7.455 7.253 7.250 7.241 7.238 7.228 7.225 7.207 7.205 7.194 7.192 7.182 7.180 7.151 7.148 7.138 7.135
2.00 2.08 2.18 1.95
fl (ppm)

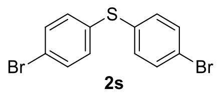

2s

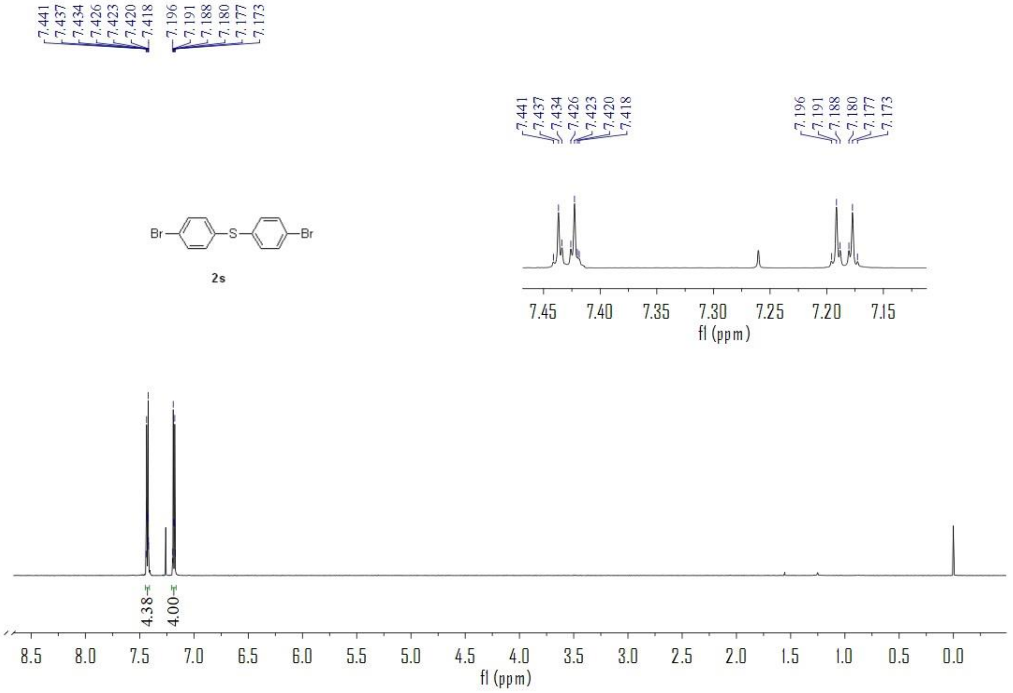

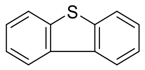

2t

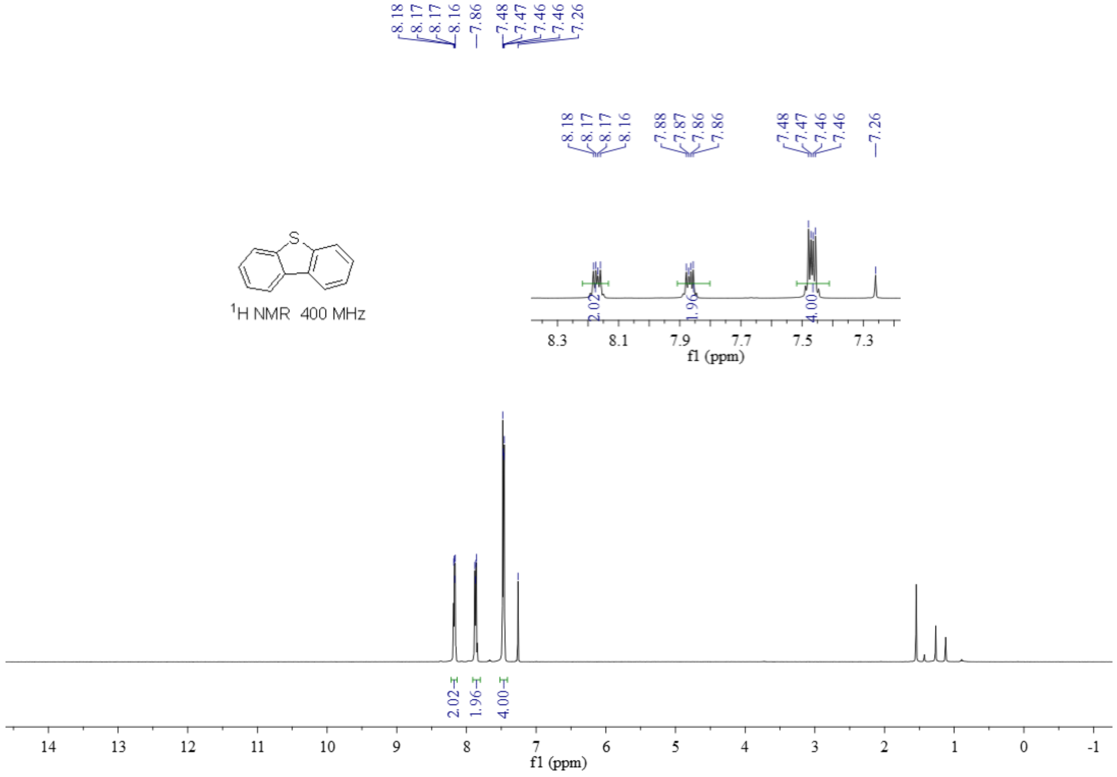

6.2 ^{1}H NMR, ^{13}C NMR and ^{19}F NMR spectra of diaryl disulfides

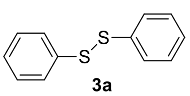
S
S

3a

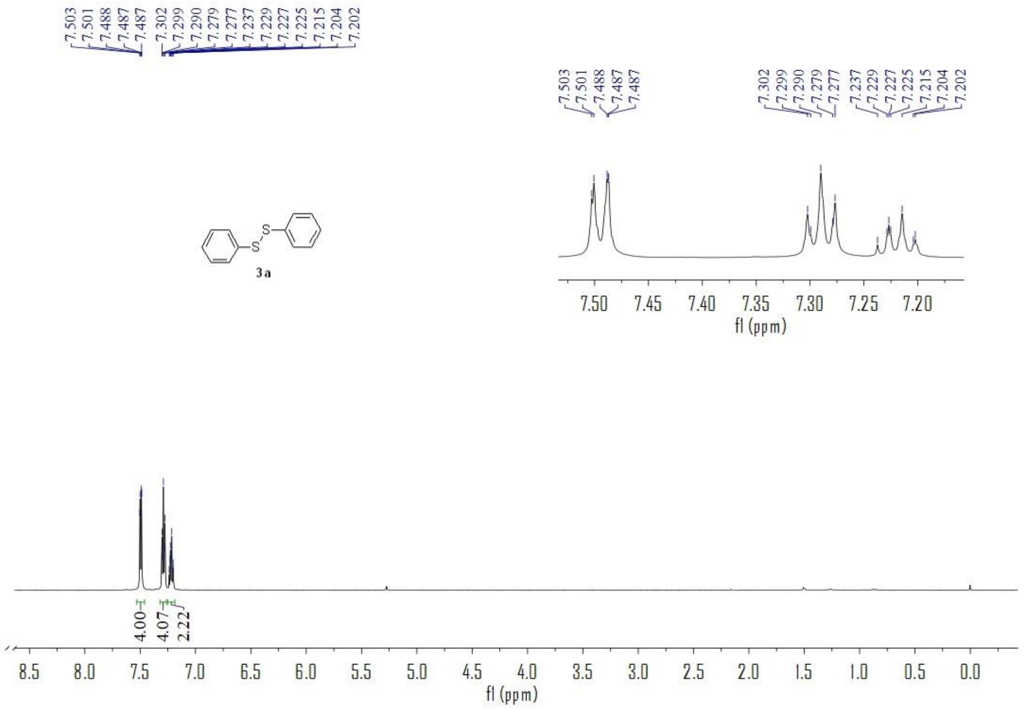
7.503
7.501
7.488
7.487
7.487
7.302
7.299
7.290
7.279
7.277
7.237
7.229
7.227
7.225
7.215
7.204
7.202
3a
4.00
4.07
2.22
fl (ppm)

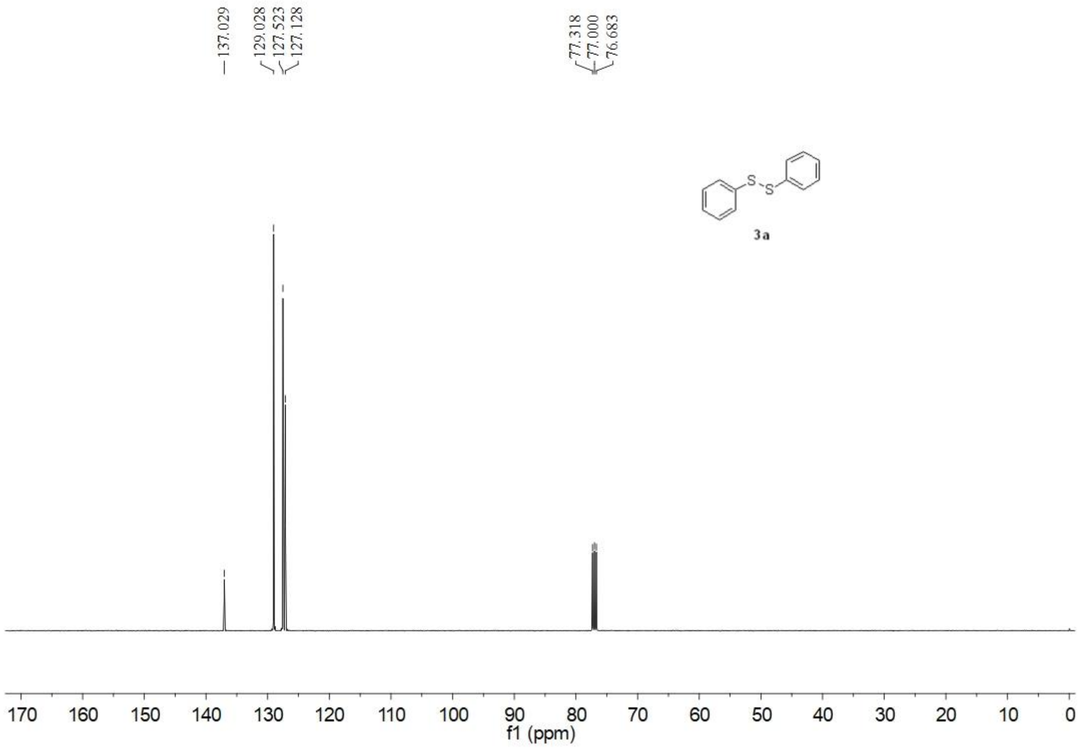
137.029
129.028
127.523
127.128
77.318
77.000
76.683
3a
f1 (ppm)

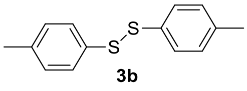
S
S

3b

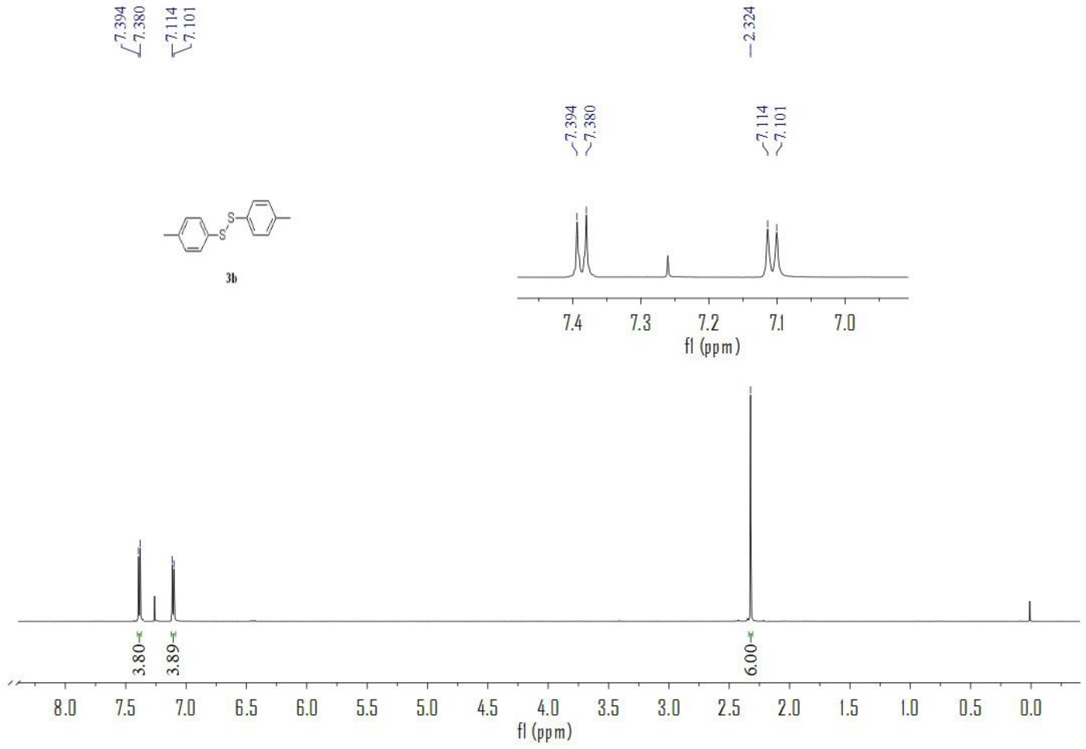

7.394
7.380
7.114
7.101
2.324
3b
7.4 7.3 7.2 7.1 7.0
f1 (ppm)
3.80
3.89
6.00
8.0 7.5 7.0 6.5 6.0 5.5 5.0 4.5 4.0 3.5 3.0 2.5 2.0 1.5 1.0 0.5 0.0
f1 (ppm)

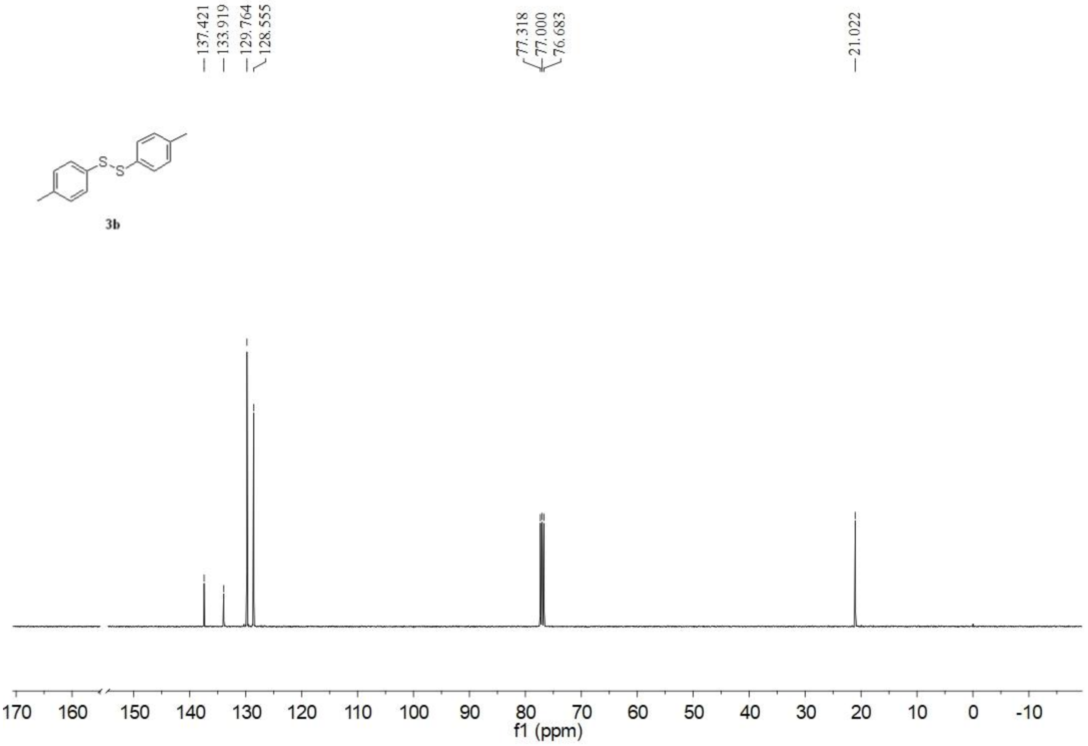

137.421
133.919
129.764
128.555
77.318
77.000
76.683
21.022
3b
170 160 150 140 130 120 110 100 90 80 70 60 50 40 30 20 10 0 -10
f1 (ppm)

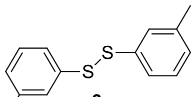

3c

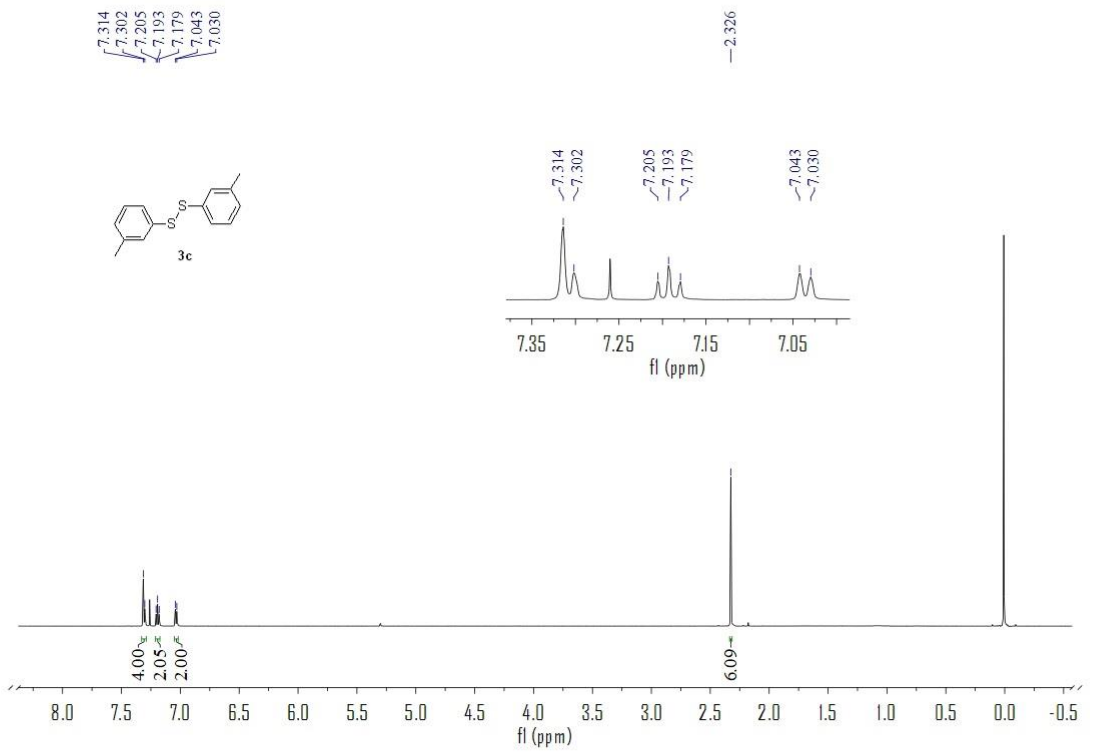
7.314
7.302
7.205
7.193
7.179
7.043
7.030
2.326
3c
7.35
7.25
7.15
7.05
f1 (ppm)
4.00
2.05
2.00
6.09
8.0 7.5 7.0 6.5 6.0 5.5 5.0 4.5 4.0 3.5 3.0 2.5 2.0 1.5 1.0 0.5 0.0 -0.5

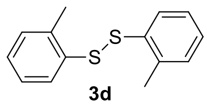

3d

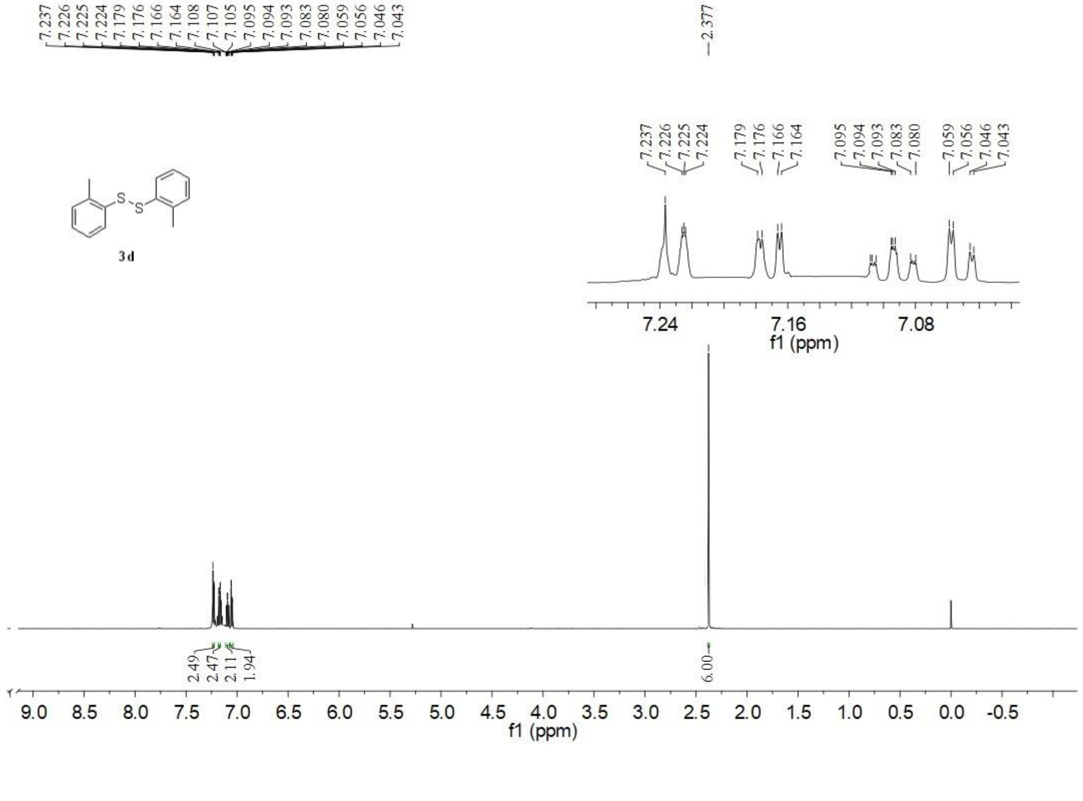
7.237
7.226
7.225
7.224
7.179
7.176
7.166
7.164
7.108
7.107
7.105
7.095
7.094
7.093
7.083
7.080
7.059
7.056
7.046
7.043
2.377
3d
7.24
7.16
7.08
f1 (ppm)
2.49
2.47
2.11
1.94
6.00
9.0 8.5 8.0 7.5 7.0 6.5 6.0 5.5 5.0 4.5 4.0 3.5 3.0 2.5 2.0 1.5 1.0 0.5 0.0 -0.5

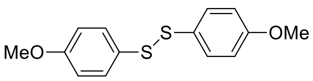
MeO
S
S
OMe

3e

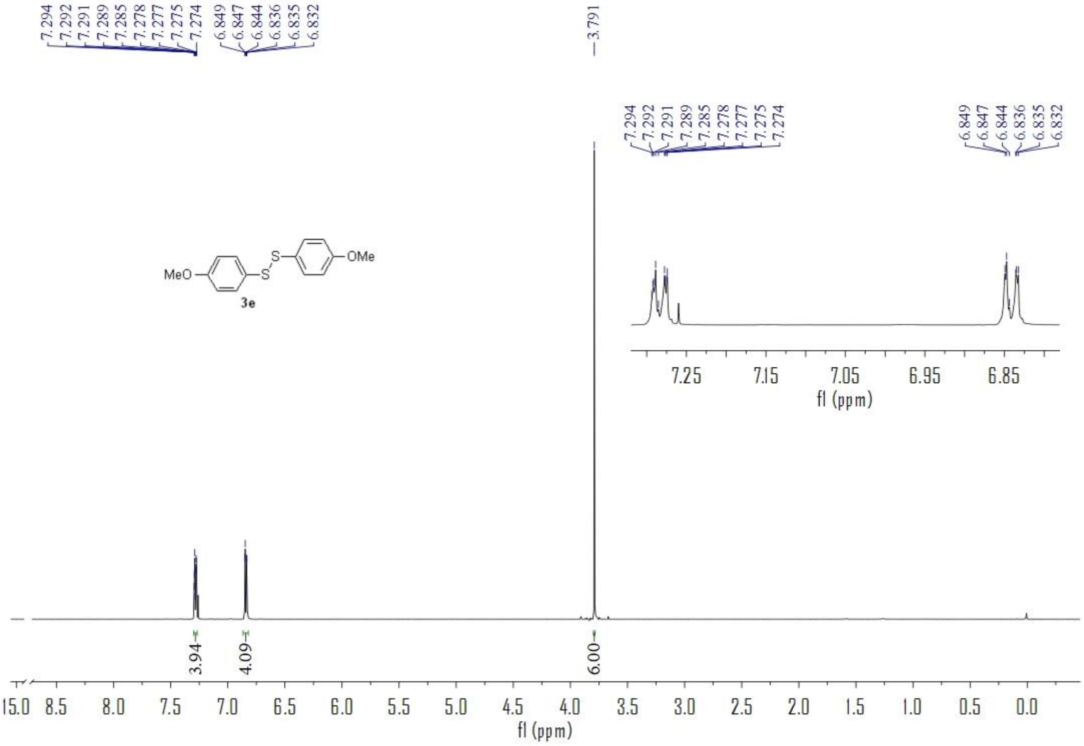
3.791
3e
3.94
4.09
6.00
fl (ppm)

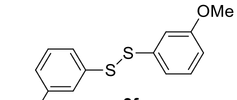
OMe
S
S
MeO

3f

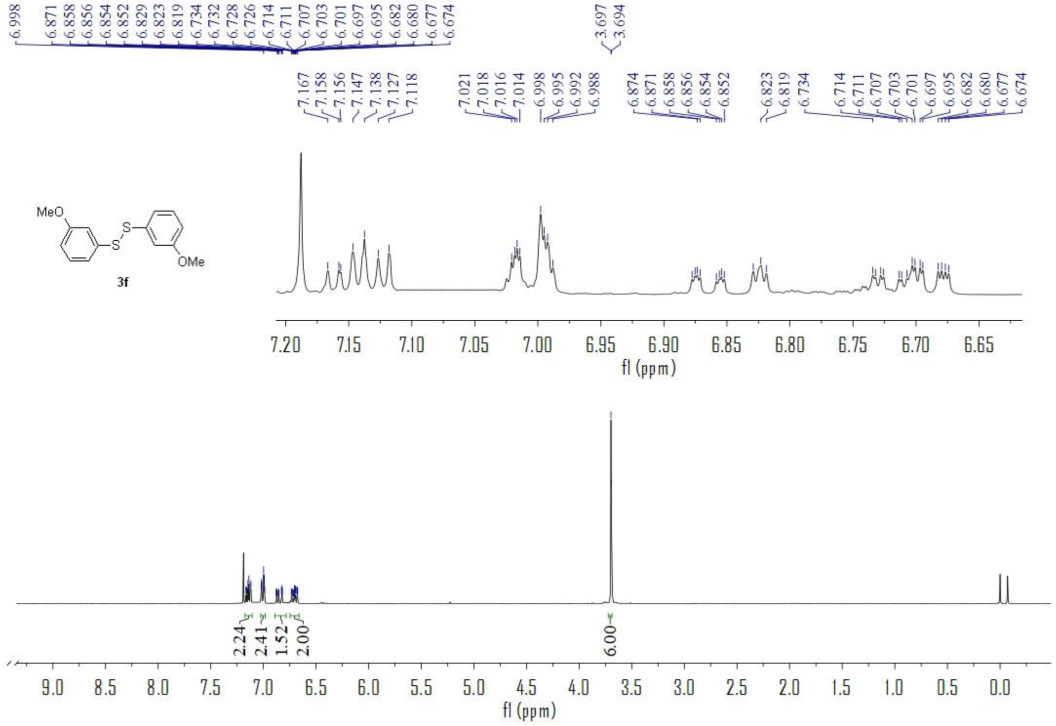
3.697
3.694
3f
2.24
2.41
1.52
2.00
6.00
fl (ppm)

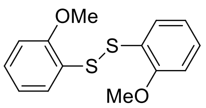
OMe
S
S
MeO

3g

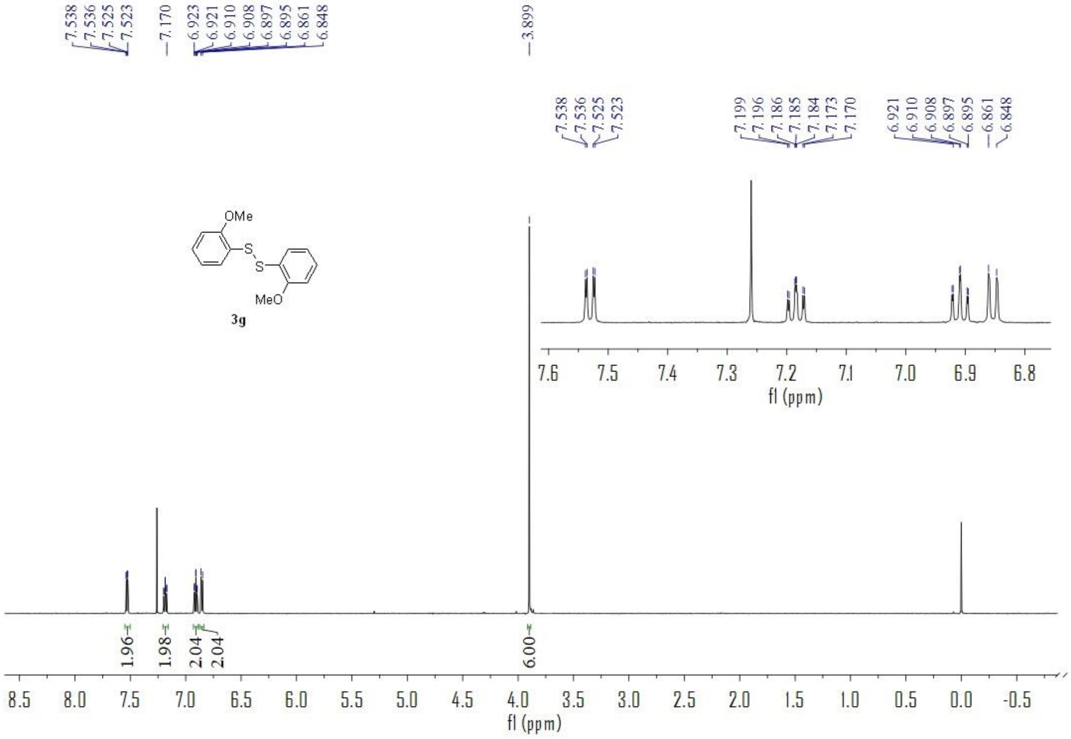
7.538
7.536
7.525
7.523
7.170
6.923
6.921
6.910
6.908
6.897
6.895
6.861
6.848
3.899
7.199
7.196
7.186
7.185
7.184
7.173
1.96
1.98
2.04
2.04
6.00
fl (ppm)
3g

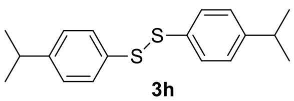
S
S

3h

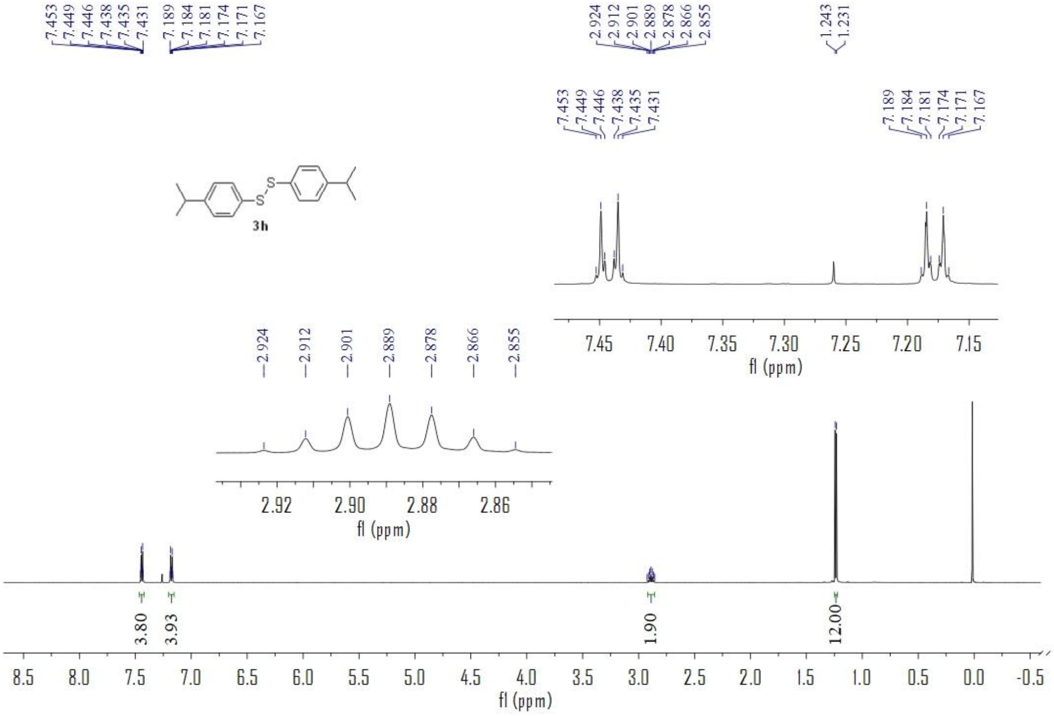
7.453
7.449
7.446
7.438
7.435
7.431
7.189
7.184
7.181
7.174
7.171
7.167
2.924
2.912
2.901
2.889
2.878
2.866
2.855
1.243
1.231
3.80
3.93
1.90
12.00
fl (ppm)
3h

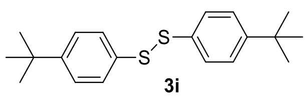

3i

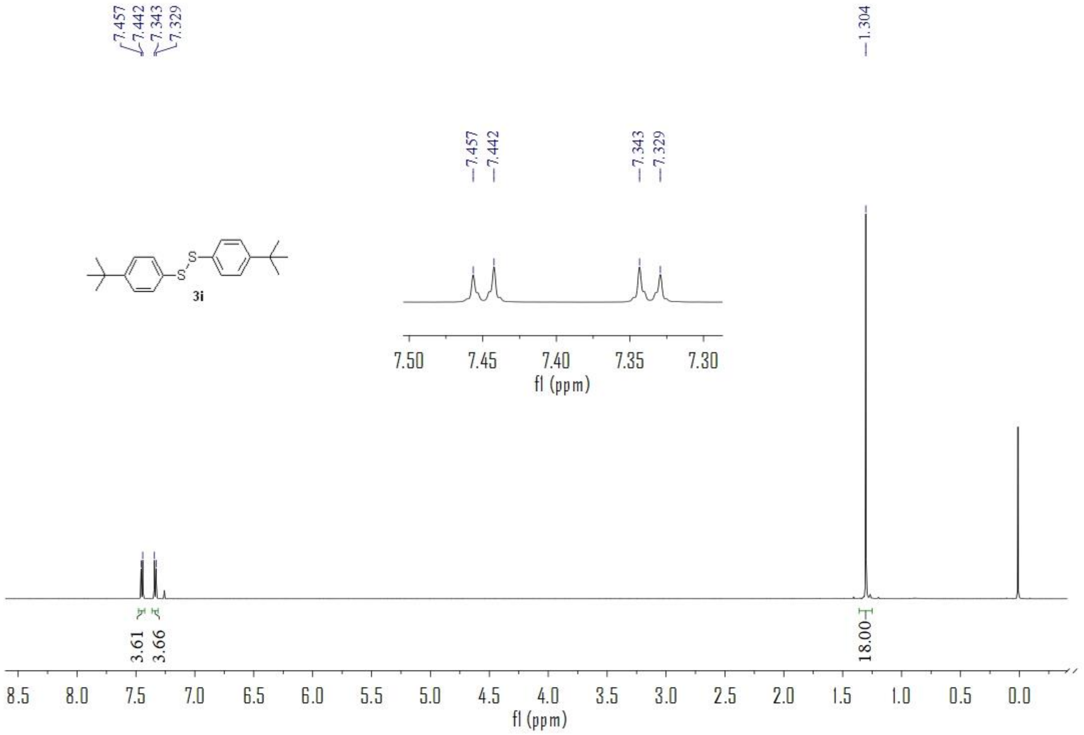

7.457
7.442
7.343
7.329
1.304
3i
7.50
7.45
7.40
7.35
7.30
fl (ppm)
3.61
3.66
18.00
8.5
8.0
7.5
7.0
6.5
6.0
5.5
5.0
4.5
4.0
3.5
3.0
2.5
2.0
1.5
1.0
0.5
0.0
fl (ppm)

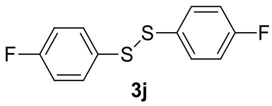

3j

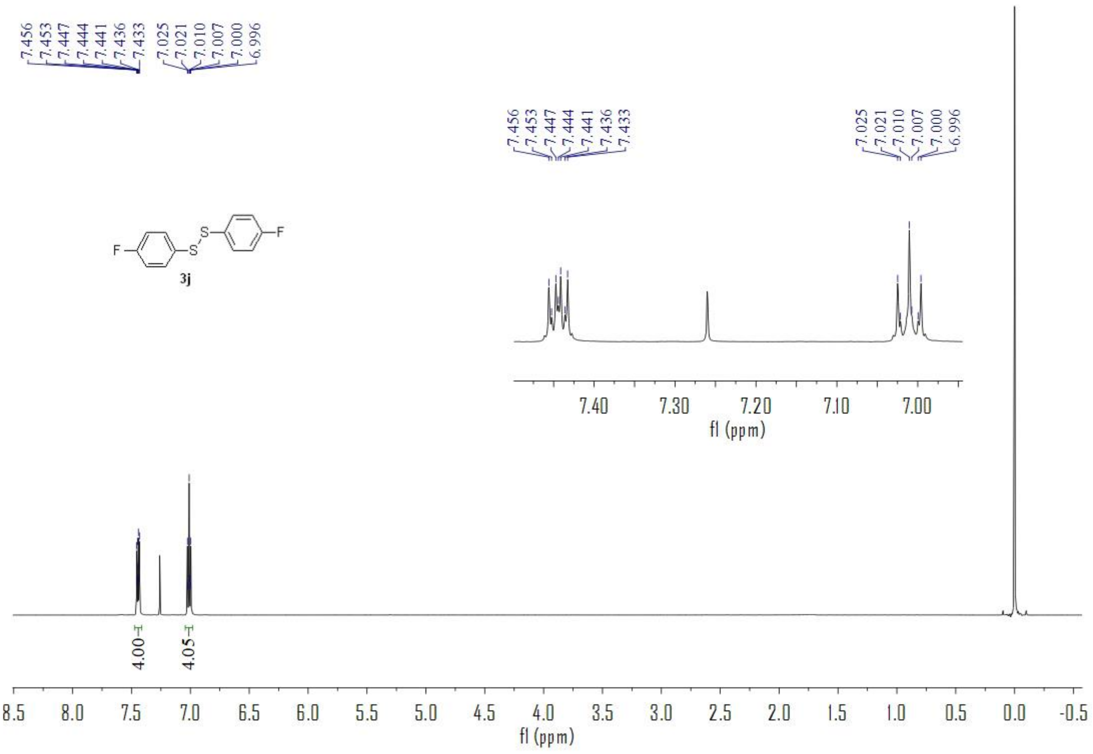

7.456
7.453
7.447
7.444
7.441
7.436
7.433
7.025
7.021
7.010
7.007
7.000
6.996
3j
4.00
4.05
8.5 8.0 7.5 7.0 6.5 6.0 5.5 5.0 4.5 4.0 3.5 3.0 2.5 2.0 1.5 1.0 0.5 0.0 -0.5
fl (ppm)
7.40 7.30 7.20 7.10 7.00
fl (ppm)

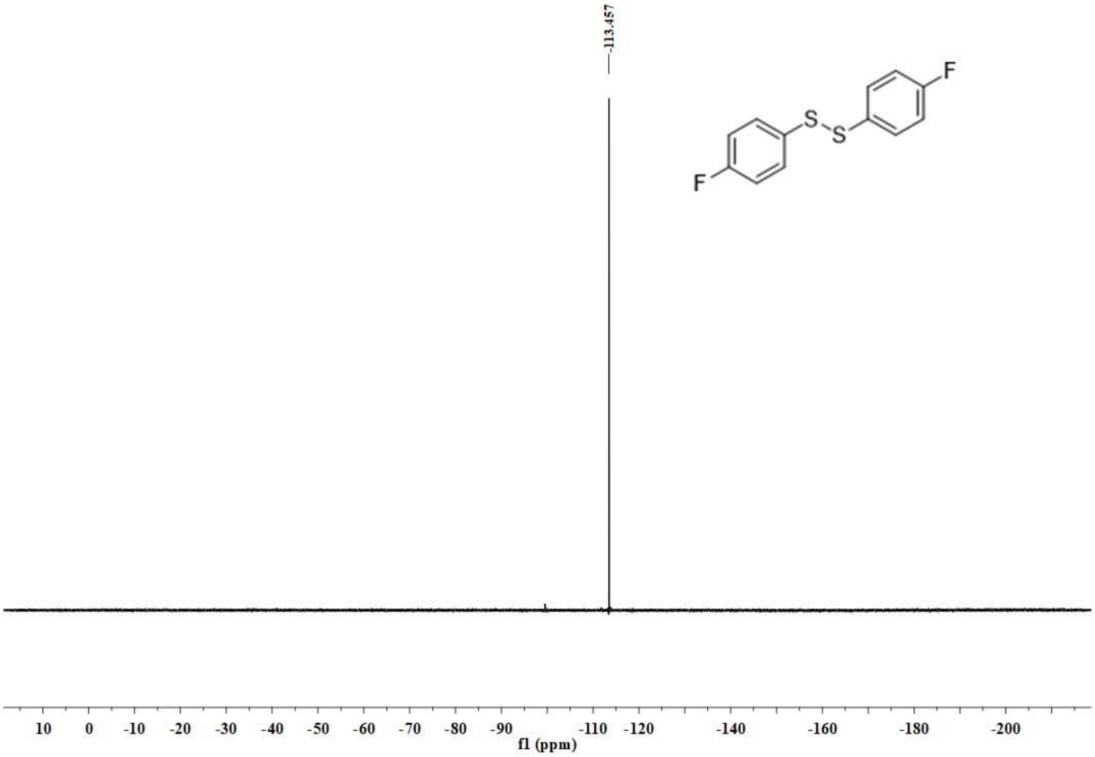

-113.457
10 0 -10 -20 -30 -40 -50 -60 -70 -80 -90 -110 -120 -140 -160 -180 -200
fl (ppm)

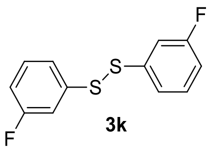
F
S
S
F

3k

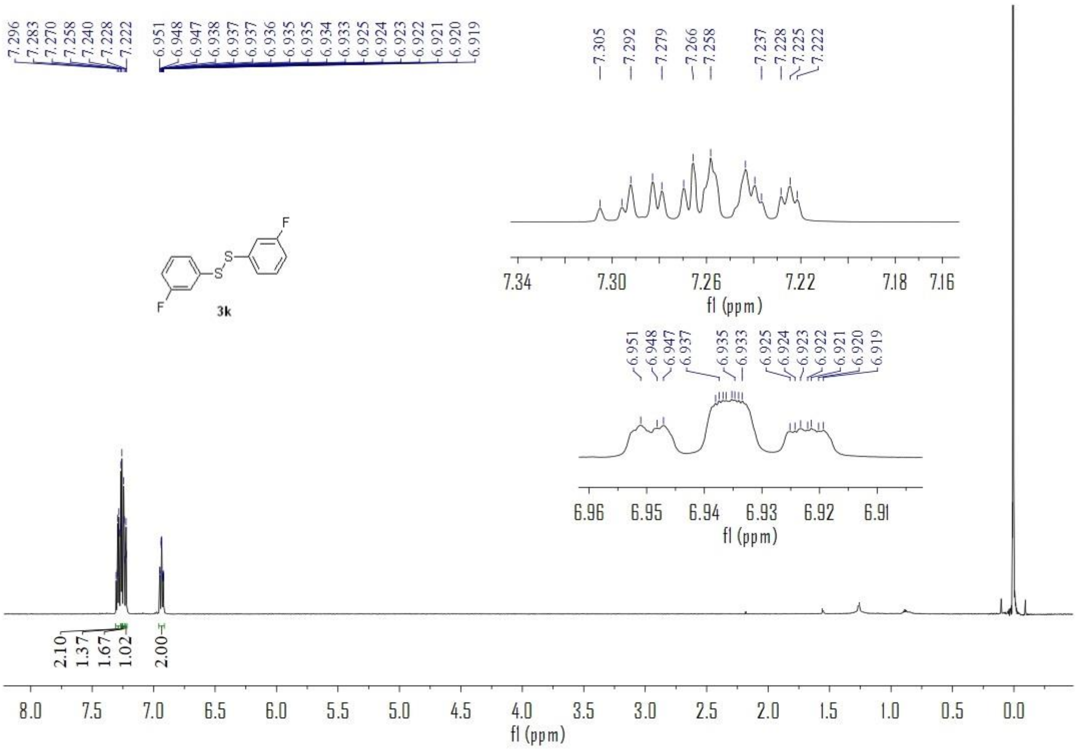
3k
7.296
7.283
7.270
7.258
7.240
7.228
7.222
6.951
6.948
6.947
6.938
6.937
6.937
6.936
6.935
6.935
6.934
6.933
6.925
6.924
6.923
6.922
6.921
6.920
6.919
7.305
7.292
7.279
7.266
7.258
7.237
7.228
7.225
7.222
2.10
1.37
1.67
1.02
2.00
fl (ppm)

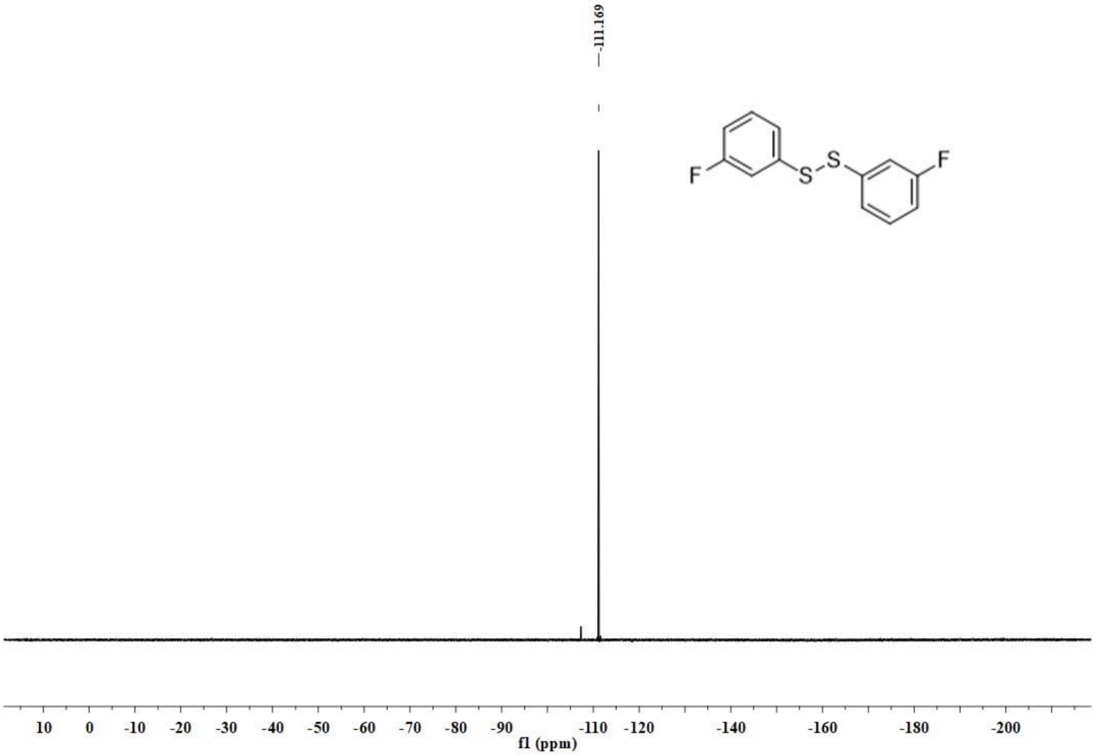
-111.169
F
S
S
F
fl (ppm)

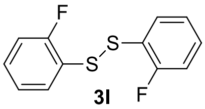
F
S
S
F

3l

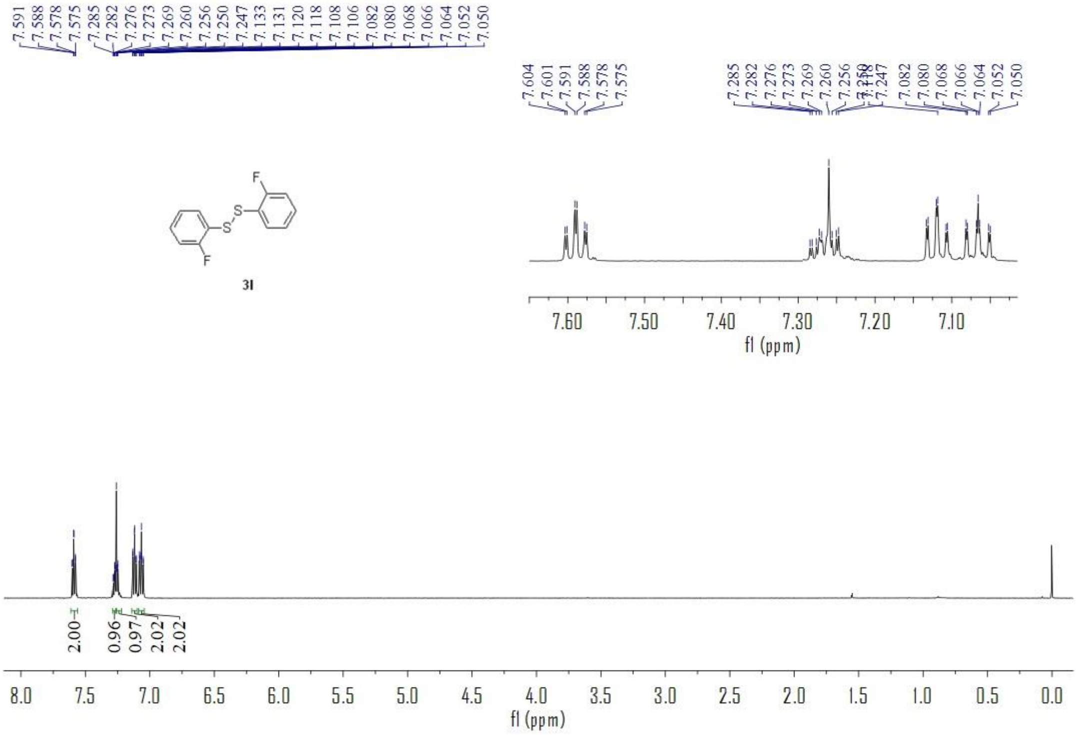
3l
7.60
7.50
7.40
7.30
7.20
7.10
fl (ppm)
2.00
0.96
0.97
2.02
2.02
8.0
7.5
7.0
6.5
6.0
5.5
5.0
4.5
4.0
3.5
3.0
2.5
2.0
1.5
1.0
0.5
0.0
fl (ppm)

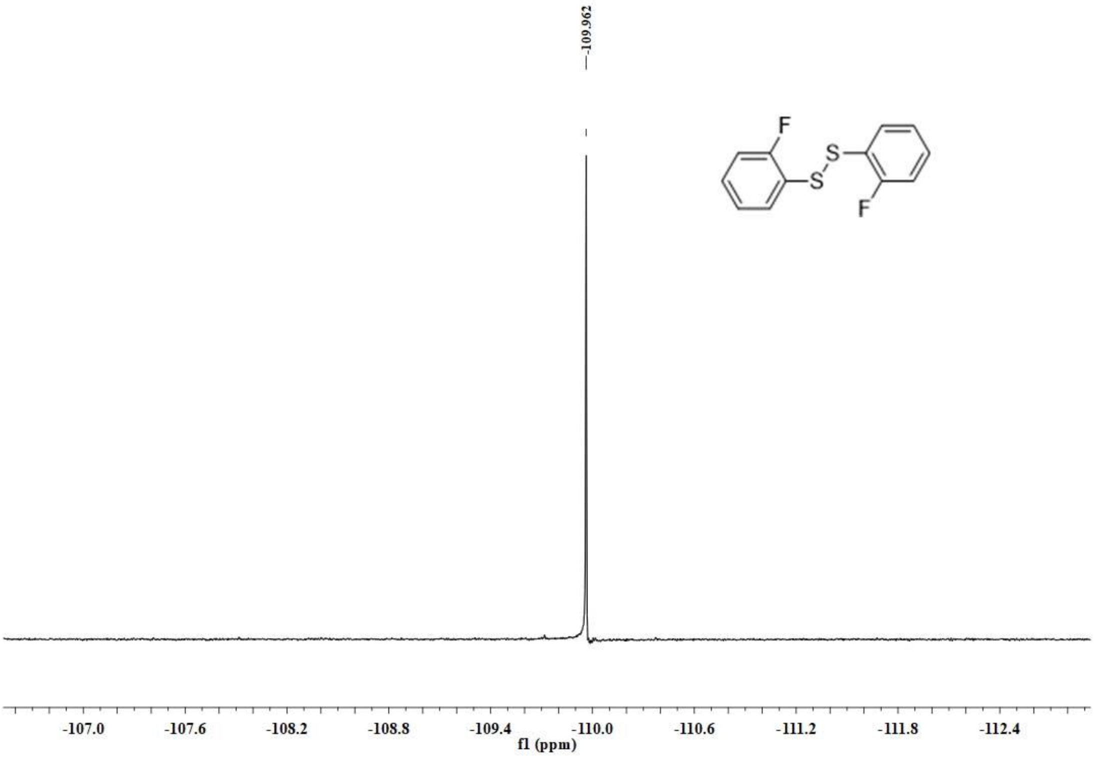
-109.962
F
S
S
F
-107.0
-107.6
-108.2
-108.8
-109.4
-110.0
-110.6
-111.2
-111.8
-112.4
f1 (ppm)

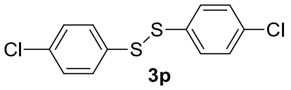

Cl
S
S
Cl

3p

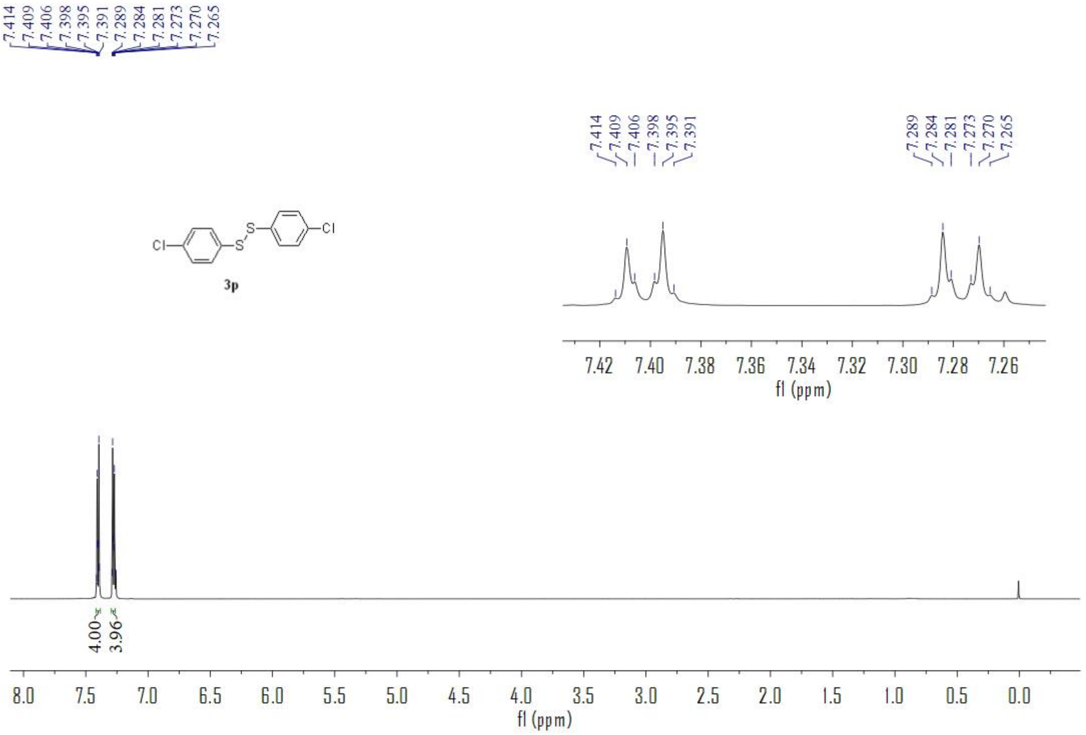

7.414
7.409
7.406
7.398
7.395
7.391
7.289
7.284
7.281
7.273
7.270
7.265
3p
7.42 7.40 7.38 7.36 7.34 7.32 7.30 7.28 7.26
f1 (ppm)
4.00
3.96
8.0 7.5 7.0 6.5 6.0 5.5 5.0 4.5 4.0 3.5 3.0 2.5 2.0 1.5 1.0 0.5 0.0
f1 (ppm)

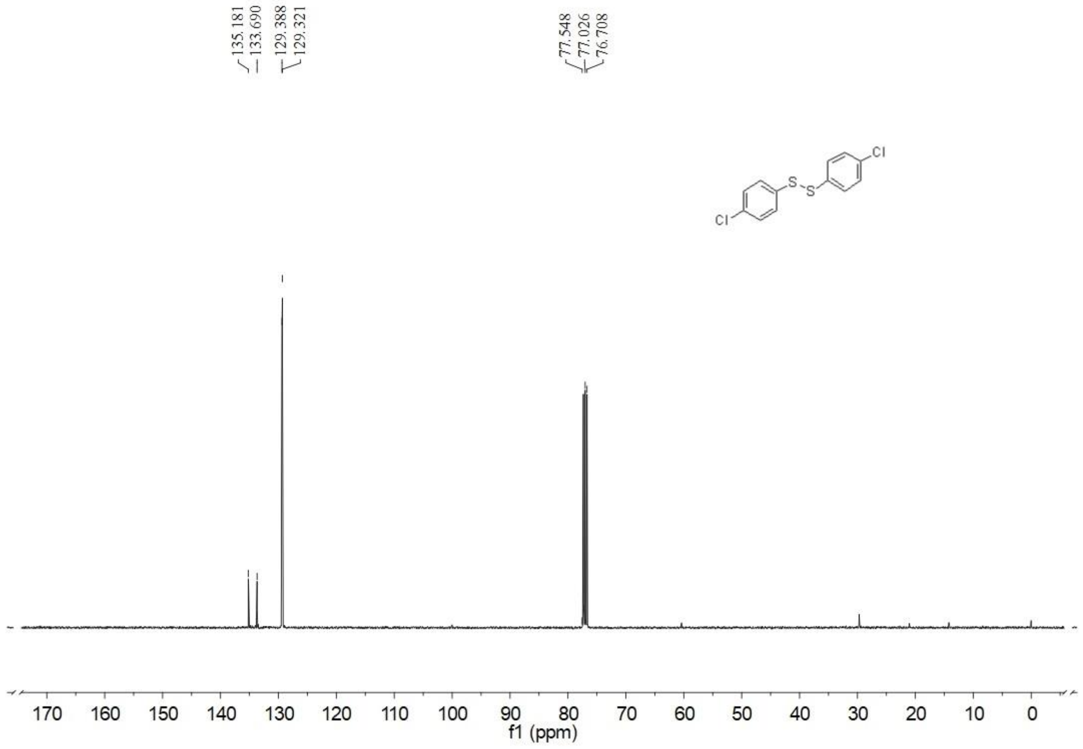

135.181
133.690
129.388
129.321
77.548
77.026
76.708
170 160 150 140 130 120 110 100 90 80 70 60 50 40 30 20 10 0
f1 (ppm)

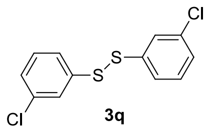

3q

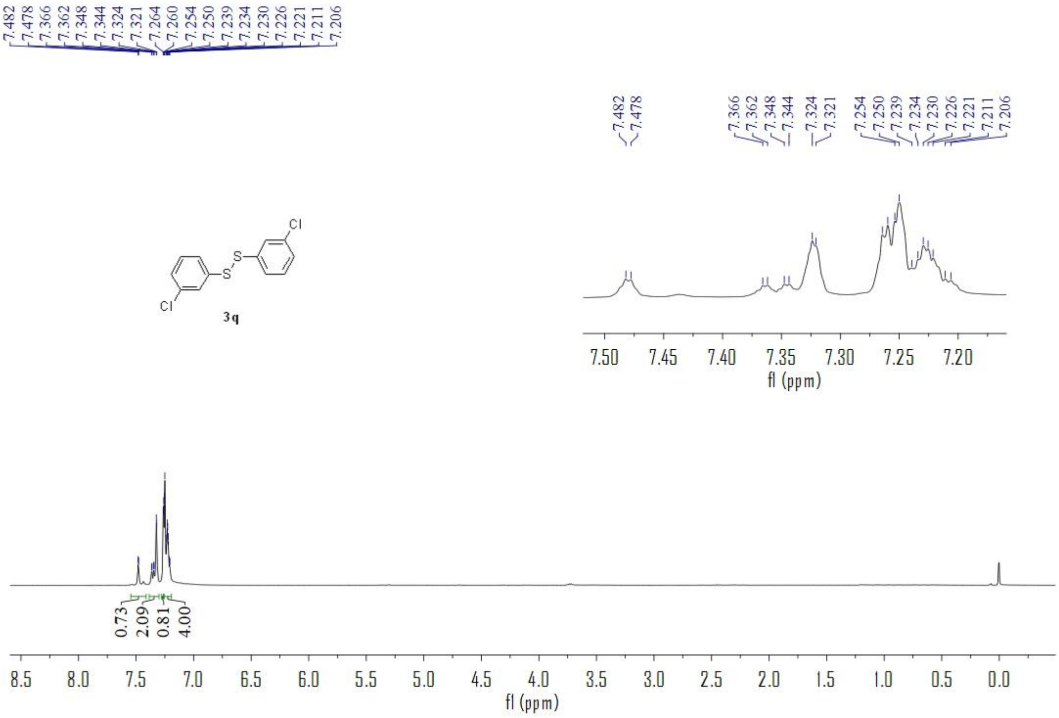

7.482
7.478
7.366
7.362
7.348
7.344
7.324
7.321
7.264
7.260
7.254
7.250
7.239
7.234
7.230
7.226
7.221
7.211
7.206
3q
0.73
2.09
0.81
4.00
fl (ppm)

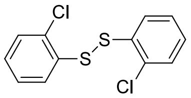

3r

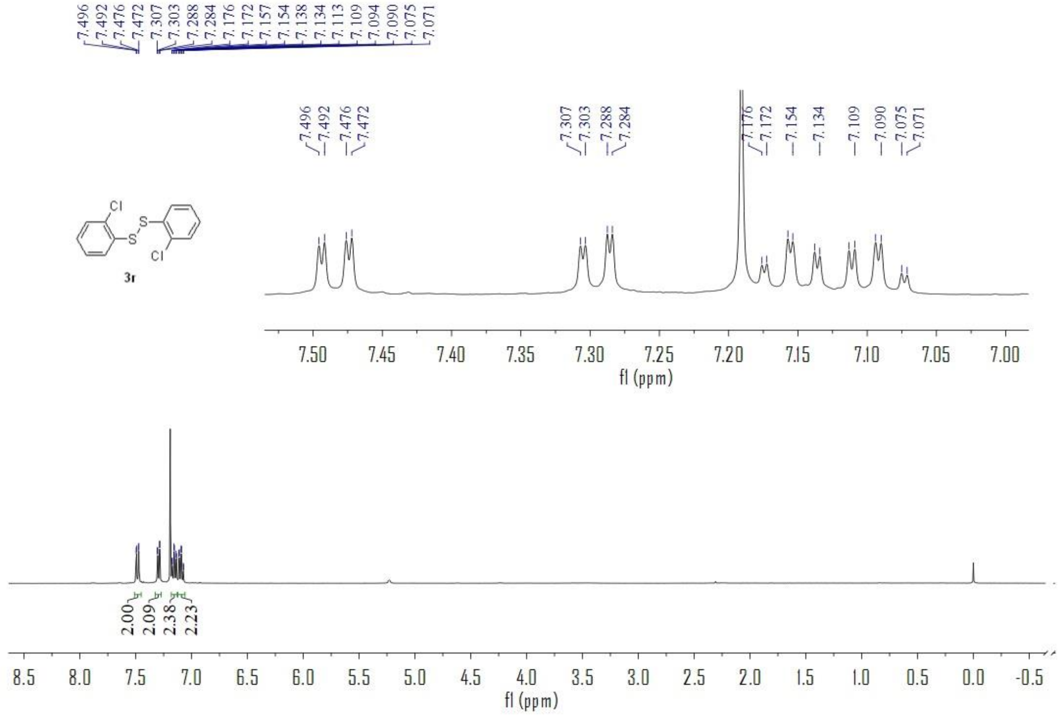

7.496
7.492
7.476
7.472
7.307
7.303
7.288
7.284
7.176
7.172
7.157
7.154
7.138
7.134
7.113
7.109
7.094
7.090
7.075
7.071
3r
2.00
2.09
2.38
2.23
fl (ppm)

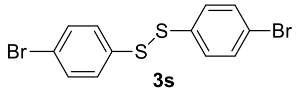
Br
S
S
Br

3s

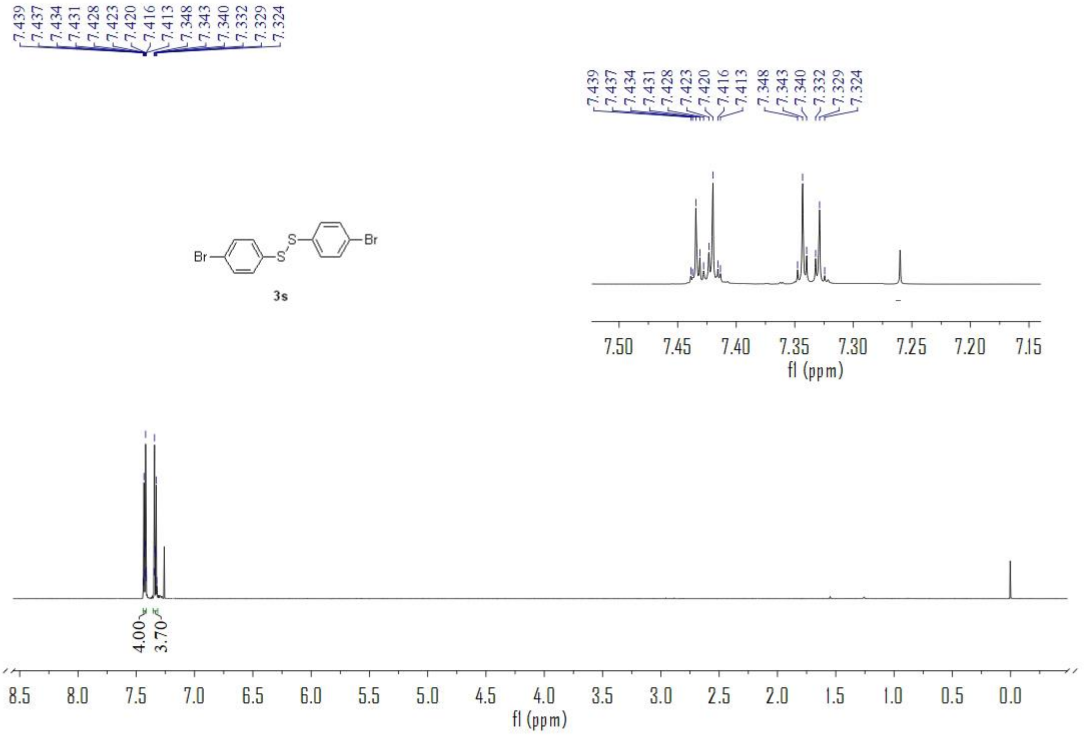
7.439
7.437
7.434
7.431
7.428
7.423
7.420
7.416
7.413
7.348
7.343
7.340
7.332
7.329
7.324
3s
4.00
3.70
f1 (ppm)

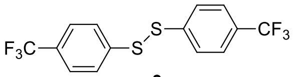
F3C
S
S
CF3

3u

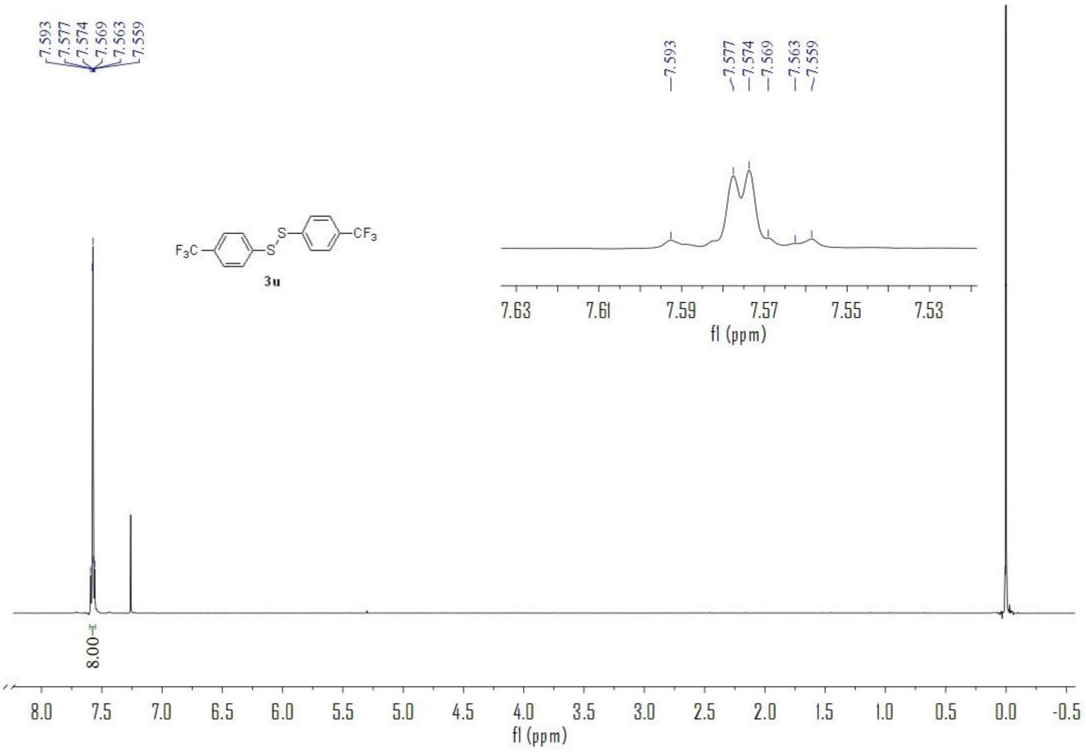
7.593
7.577
7.574
7.569
7.563
7.559
3u
8.00
f1 (ppm)

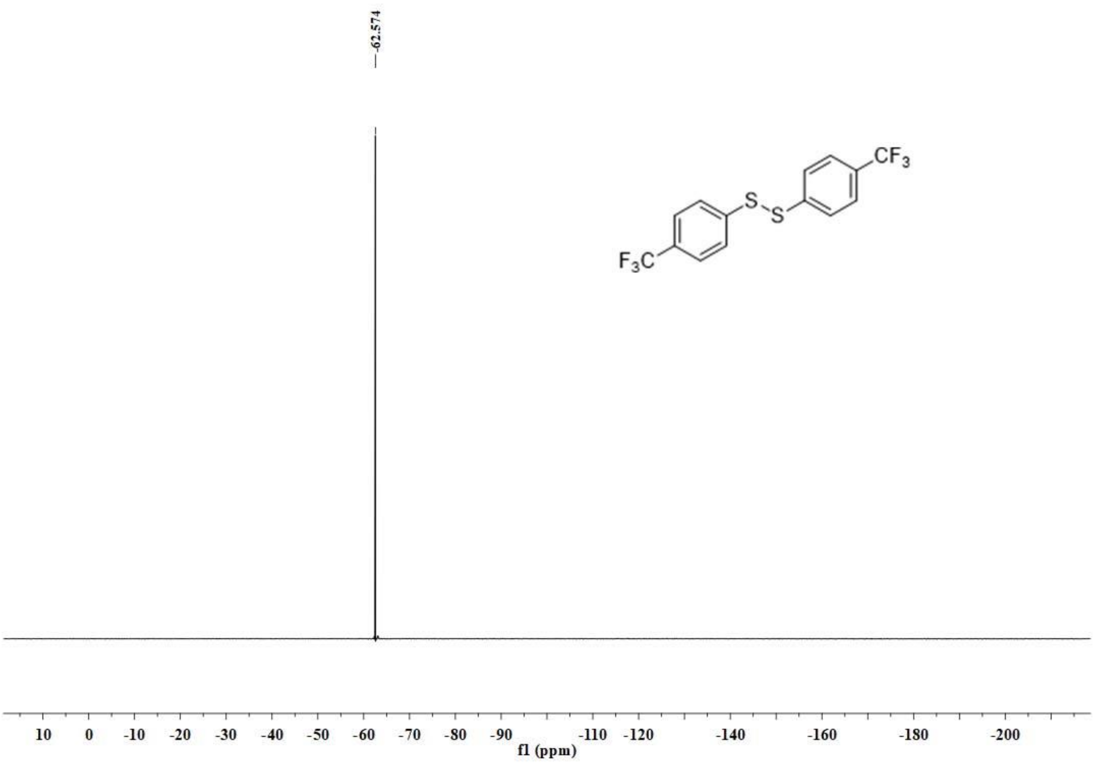
-62.574
CF3
F3C
S
S
10 0 -10 -20 -30 -40 -50 -60 -70 -80 -90 -110 -120 -140 -160 -180 -200
fl (ppm)

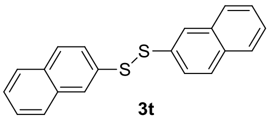

3t

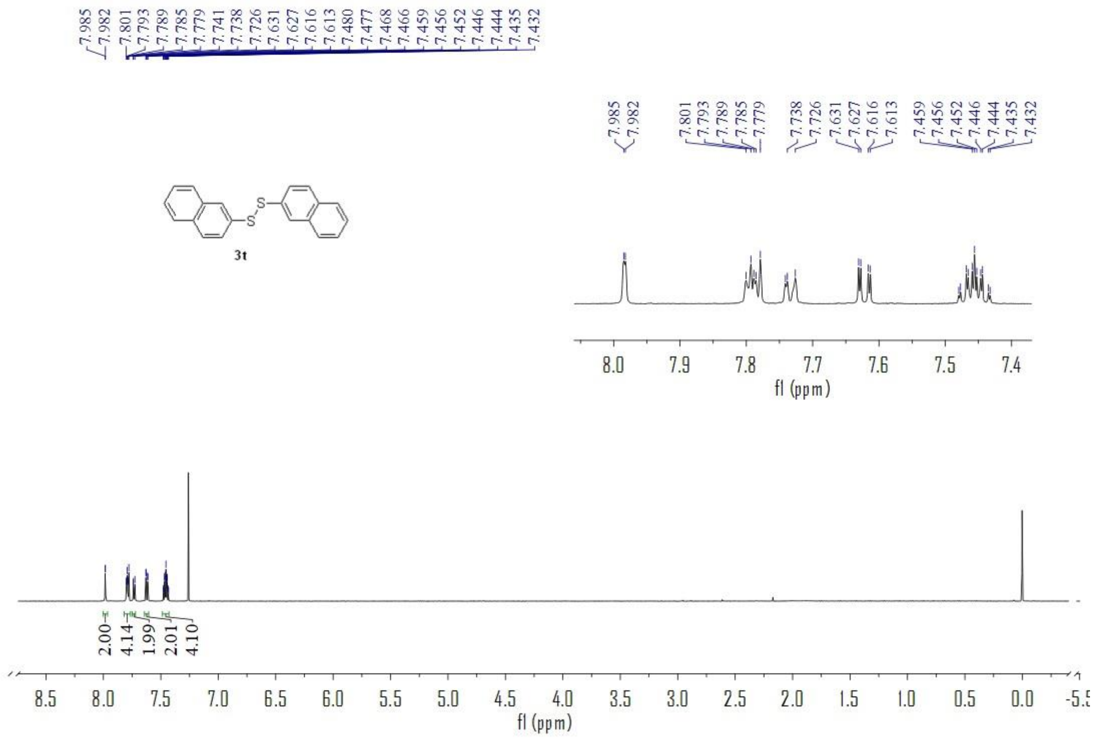
7.985
7.982
7.801
7.793
7.789
7.785
7.779
7.741
7.738
7.726
7.631
7.627
7.616
7.613
7.480
7.477
7.468
7.466
7.459
7.456
7.452
7.446
7.444
7.435
7.432
3t
8.0 7.9 7.8 7.7 7.6 7.5 7.4
fl (ppm)
2.00
4.14
1.99
2.01
4.10
8.5 8.0 7.5 7.0 6.5 6.0 5.5 5.0 4.5 4.0 3.5 3.0 2.5 2.0 1.5 1.0 0.5 0.0
fl (ppm)

HPLC analysis of diphenyl sulfide and diphenyl disulfide

Analytical and preparative HPLC measurements were performed on Shimadzu Essentia LC-16 with DGU-20A detector using C18 column (250 x 4.6 mm, 5 μm). Water and acetonitrile containing 0.1%TFA were used as the mobile phase, at a flow rate of 0.7 mL/min.

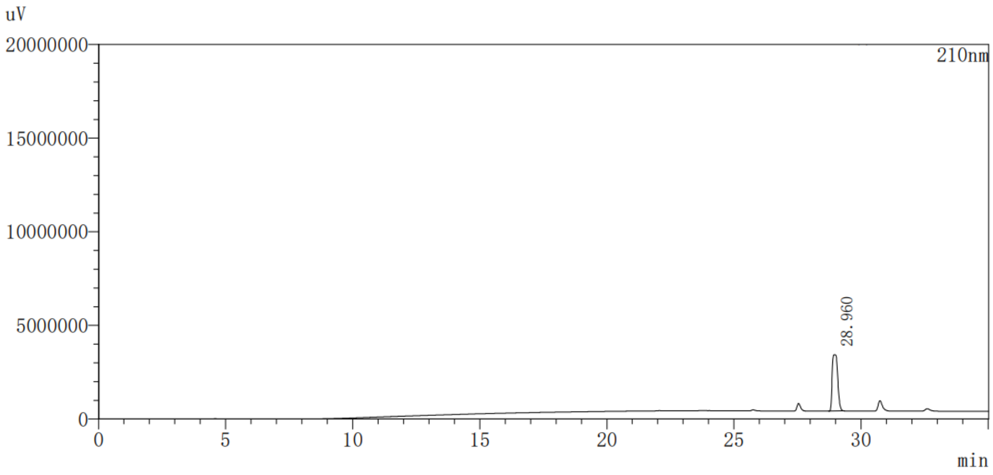

Scheme 1. HPLC analysis of diphenyl sulfide

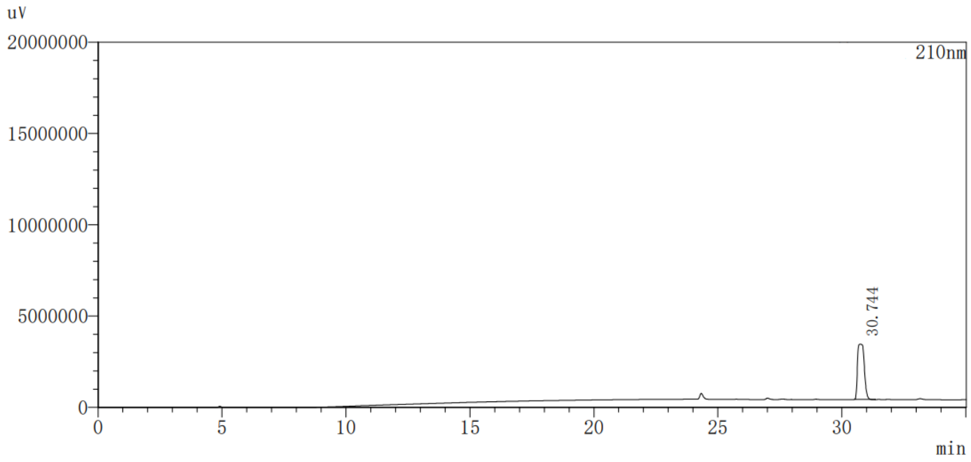

Scheme 2. HPLC analysis of diphenyl disulfide

Entry	Retention time
1	28.960
2	30.744

IR for diphenyl sulfide and diphenyl disulfide

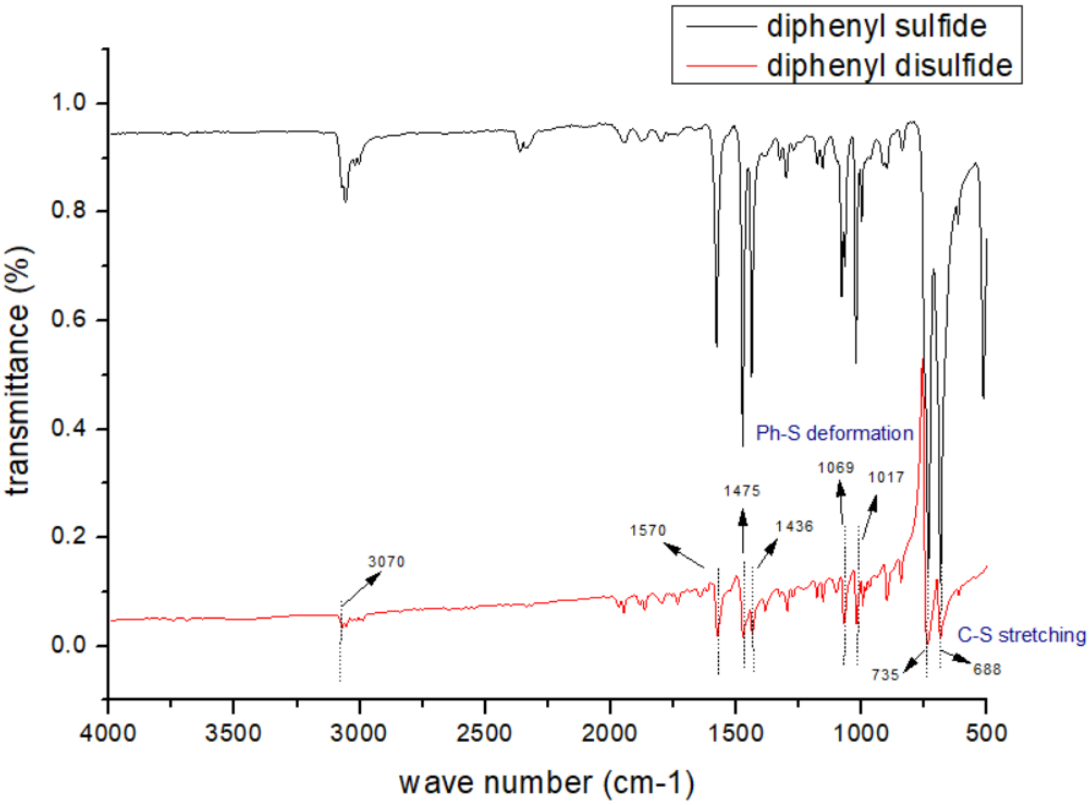

IR: 3070 (C-H stretching vibration), 1570, 1475, 1436 (C-C stretching vibration), 1069, 1017 (Ph-S deformation vibration), 735, 688 (C-S stretching vibration) cm^{-1}
